# Patterns of cognitive and motor decline in Alzheimer’s Disease (AD) and ageing in healthy populations

**DOI:** 10.1007/s40520-026-03327-1

**Published:** 2026-01-31

**Authors:** Maddalena Beccherle, Stefania Amato, Elena Facci, Georgeta Stefanescu, Sara Bertagnoli, Vincenzo Di Francesco, Giorgia Fontana, Giuseppe Gambina, Valentina Moro

**Affiliations:** 1 NPSY‑Lab.VR, Department of Human Sciences, University of Verona, Lungadige Porta Vittoria, 17, Verona, 37129 Italy; 2 Verona Memory Center - CEMS, Via Lorenzo Fava, 2, Verona, 37139 Italy; 3 Associazione Familiari Malati di Alzheimer, Via Guglielmo Marconi, 21, Verona, 37122 Italy; 4 Social and Cognitive Neuroscience Laboratory, Department of Psychology, University La Sapienza, Via dei Marsi, 78, Rome, 00185 Italy; 5 Geriatric Unit A, Azienda Ospedaliera Universitaria Integrata di Verona, Via Goffredo Mameli, Verona, 37126 Italy; 6 IRCCS Sacro Cuore Don Calabria, Negrar, Via Don A. Sempreboni, 5, Verona, 37024 Negrar Italy

**Keywords:** Cognitive and motor decline, Psychomotor abilities, Alzheimer’s Disease, Healthy ageing, Nutritional status

## Abstract

**Background:**

Various patterns may apply to an individual’s health-span, with quality of life deriving from a balance between physical conditions, motor and cognitive abilities (i.e. psychomotor capabilities).

**Methods:**

In this study, the Italian version of the Éxamen Geronto-Psychomoteur was administered to a sample of Alzheimer’s Disease (AD) patients (*n* = 94) and a group of healthy older adults (*n* = 333) to compare the patterns of psychomotor decline in pathological and physiological ageing. Three domains were considered to integrate bodily and cognitive dimensions: cognitive functions, motor abilities, and muscular tone alterations (physical constraints). Potential correlations with general cognitive functioning, autonomy in daily life, mood and nutritional status were also investigated.

**Results:**

A correlation between cognitive, motor and physical dimensions is confirmed, and the results show that the patterns relating to healthy and pathological ageing are not only quantitatively but also qualitatively different. Besides the cognitive functions, the deterioration in AD also affects the physical components, precociously. Specifically, hypertonia may be present since the initial phases of illness. In healthy subjects, body representations decline early, while verbal memory, temporal and space representation resist over time. Malnutrition correlates with hypertonia in AD and with a reduction in daily life abilities in healthy people.

**Conclusions:**

The results highlight the importance of adopting an integrated psychomotor approach in the screening, diagnosis and treatment of ageing and AD to investigate early motor and bodily indicators, which are often not fully considered in clinical practice.

**Supplementary Information:**

The online version contains supplementary material available at 10.1007/s40520-026-03327-1.

## Introduction

Average human life expectancy has increased over the last few decades in industrialised countries. This trend will probably continue, given the significant, widespread improvements in health, food and lifestyle conditions [1]. Two complementary processes derive from this situation: on the one side, the pathologies associated with ageing are more diffuse, leading to increased health costs; on the other, the development of new concepts relating to the quality of ageing (known as “health-span”). This term refers to the average number of years a person remains healthy and is associated with wellbeing rather than the mere absence of disease [2]. This reflects a relative change in perspective, also demonstrated by the World Health Organisation’s definition (2020), according to which healthy ageing is the process of “developing and maintaining the functional ability that enables wellbeing in older age” [3].

Accordingly, the concept of “functional ability” becomes crucial in healthy as well as in pathological ageing, since it enables wellbeing and promotes quality of life [4–6]. Functional ability derives from a balance between physical condition, motor functions and cognitive abilities, in other words, an individual’s psycho-motor capabilities. This perspective supersedes traditional theories positing that mental activity relies on higher-order cognition and emotions, and that somatosensory and motor bodily functions are purely instrumental components of cognitive functions (e.g. “the sandwich model”, [7]). Conversely, it also embraces the idea that cognition is embodied, and thus that individual abilities are the result of a complex interplay between the body and the brain interacting with the surrounding environment [8, 9]. The same applies also to ageing processes and decline, where cognitive and body-related aspects interact constantly [10]. In recent years, embodied cognition approaches have attracted the interest of many disciplines, such as Psychology [11–13], Robotics [14], Artificial Intelligence [15] and Neuroscience [16–18]. Furthermore, these theoretical frameworks have impacted how neurological diseases are approached [19], meaning that growing attention is being paid to both the brain and body axes, which are now increasingly considered as mutually influencing each other rather than separate domains. Clinical conditions which were traditionally considered as purely motor disorders (e.g. Parkinson’s Disease, spinal cord injury, chronic pain) are now also studied in their cognitive aspects (e.g [20–22]). and other pathologies typically considered as conditions mainly affecting cognitive domains (e.g. dementias), are now investigated also in their sensory-motor, bodily components [23–26]. Unfortunately, in clinical practice, few instruments are available to assess the various sensory-motor and cognitive functions in an integrated way to provide a psychomotor profile [27].

The present study aimed to compare the patterns of ageing in patients with Alzheimer’s Disease (AD) and a group of age-matched, healthy participants without any history of brain disease. Cognitive and sensory-motor functions were assessed by a screening tool, the *Examen Géronto-Psychomoteur* (EGP) [28]. This was developed in France to assess psychomotor skills in ageing and constitutes a first screening for pathological symptoms of cognitive decline. The tool was initially created by Michel and colleagues, [28] and then translated and validated in Portugal and Uruguay [29, 30], Lebanon [27] and Brazil [31]. In the Portuguese version, the authors carried out a confirmatory factor analysis on the EGP items, according to which three main factors emerged [29, 30]. *Cognitive prevalence* concerns the cognitive domain and involves Vigilance (i.e. sustained attention), Communication, Space representation, Temporal orientation, Verbal and Perceptual memory, and Praxis. This component also comprises functions associated with the body, such as Fine motricity of the upper limbs, Knowledge and recognition of Body parts, and Perception. *Motor prevalence* includes items related to Static and Dynamic Balance and Fine Motricity in lower limbs. *Physical Constraints* are measured by means of the mobilisation of specific joints in the upper and lower limbs and a clinical assessment of changes in muscle tone.

The primary aim of the study was to compare the patterns of psychomotor ageing in pathological and healthy populations and to identify early psychomotor markers of disease, through the administration of the EGP to AD patients and a large group of neurologically healthy older adults (> 60 years). The second aim of the research was to examine the progression of psychomotor and functional decline across AD by comparing AD subgroups suffering from varying degrees of impairment. Finally, in light of recent evidence suggesting a complex interplay between functional decline, mood, nutritional status and autonomy in daily life activities [32–34], the presence of potential associations between these variables in our sample was investigated.

## Methods

### Participants

427 participants (≥ 60 years) were recruited over a period of 8 years. 94 of them had been diagnosed with Alzheimer’s Disease (AD), while the other 333 were neurologically healthy. Healthy older adults were recruited among experimenters’ acquaintances and at the Geriatric Unit of a public hospital in Verona. AD patients were enrolled from a centre for Alzheimer’s Disease (i.e., an association of AD patients and caregivers) and a memory clinic in Verona. The diagnosis of AD was made by an expert neurologist, based on neurological and neuropsychological assessments, neuroimaging biomarkers (MRI or PET), and cerebrospinal fluid, when requested, according to McKhann’s and Dubois’ criteria (for “prodromal” AD) [35, 36]. Additional patient enrolment criteria included preserved verbal comprehension and the absence of behavioural disorders. No limitations in terms of Mini-Mental State Examination (MMSE) scores, [37] and education were applied. Patients with a history of head injury, psychiatric disorders, other neurological diseases, or severe sensory-motor deficits which prevent the task execution were excluded. Based on the Starkstein’s classification, i.e. the degree of impairment determined according to their MMSE scores (age and education-corrected), the patients were clustered into four classes of severity (< 15 = Severe; 15-19.50 = Moderate; 19.51–24.50 = Mild; 24.51-30 = Very mild—including ‘‘prodromal’’— AD, [38]).

333 neurologically healthy participants (60 to 90 years) were recruited according to the same criteria used for the AD patients. The MMSE was administered to exclude the presence of undiagnosed cognitive deficits (see SM1 for details). The HC sample was then clustered into seven groups according to age (60–64; 65–69; 70–74; 75–79; 80–84; 85–89; > 90 years) to investigate the patterns of psychomotor decline in healthy ageing (Supplementary Materials 4, SM 4). The performance of a subgroup of 132 healthy participants, matched for age (range: 75–84 years) with the AD sample, was compared with the patients (Table 1).

**Table 1:** Demographic characteristics of the Alzheimer’s Disease (AD) groups (Very mild: MMSE = 30-24.51; mild AD: MMSE = 24.50-19.51; moderate AD: MMSE = 19.50-15; severe AD: MMSE < 15) and of the age-matched control group (HC, age range: 75-84 years)

	HC	Very mild AD	Mild AD	Moderate AD	Severe AD	Group comparisons
**N**	132	19	30	24	16	
**Female/male**	88/ 44	11/8	17/13	12/12	10/ 6	
**Age (y)** (M ± SD)	79.02 ± 2.67	77.05 ± 6.02	79.67 ± 5.2	80.33 ± 6.77	80.25 ± 5.73	n.s.
**Education** (M ± SD)	8.08 ± 4.13	7.37 ± 3.06	7.47 ± 3.27	6.5 ± 3.76	7.56 ± 4.73	n.s.
**Physically active/non-active**	87/40	3/12	13/13	3/20	5/8	* °
**MMSE** (M ± SD) (Folstein et al., 1975) [37]	28.26 ± 1.65	25.88 ± 1.4	22.19 ± 1.58	17.59 ± 1.45	10.15 ± 3.6	* + ° ^
**ADL**^**$**^ (Mdn [range]) (Katz et al., 1963) [41]	0 [0-3]	0 [0-6]	0 [0-6]	1 [0-6]	3 [0-6]	* + ° ^
**IADL**^**$**^ (Mdn [range]) (Lawton & Brody, 1969) [42]	0 [0-4]	2 [0-7]	2 [0-7]	4 [0-7]	4 [0-8]	* + ° ^
**MNA-SF**^**$**^ (M ± SD) (Rubenstein et al., 2001) [39]	11.88 ± 2.49	11.43 ± 1.81	11.11 ± 2.25	10.75 ± 2.26	10.75 ± 1.5	n.s.
**GDS**^**$**^ (M ± SD) (Yesavage et al., 1982) [40]	2.42 ± 2.21	4.7 ± 4.99	3.95 ± 2.56	2.88 ± 1.96	3.67 ± 3.5	°

MMSE: mini-Mental state Examination; ADL: activity of daily living (number of lost abilities); IADL: instrumental activity of daily living (number of lost abilities); MNA-SF: mini nutritional Assessment-Short form (cut-off ≤10); GDS: geriatric depression scale (cut-off ≥ 6). ^$^= missing data: ADL (5.9%), IADL (9.5%), MNA -SF (29.9%), and GDS (18.1%). Significant differences between the AD groups and HC. Legend: n.s. = not significant; * = Very mild AD vs. HC; + = mild AD vs. HC; ° = moderate AD vs. HC; ^ = severe AD vs. HC

### Preliminary examination

In addition to demographical data, information about the participants’ social conditions (i.e. household composition, employment status) and physical activities was collected. Furthermore, they filled out two questionnaires investigating their nutritional status (Mini Nutritional Assessment- MNA-SF, [39]) and mood (Geriatric Depression Scale, GDS, [40]). Finally, autonomy in daily life activities was assessed by means of the Activities of Daily Living Scale (ADL, [41]) and the Instrumental Activities of Daily Living Scale (IADL, [42]). Whenever a participant could not provide this information, the caregiver was interviewed.

### The assessment of cognitive and psychomotor functions: the *Examen Géronto-Psychomoteur* (EGP)

The EGP [28] consists of 17 items that assess the following dimensions (Table 2): static and dynamic balance; joint mobilisation; praxis; fine motor skills of upper and lower limbs; knowledge of body parts; vigilance; perception; verbal and perceptual memory; spatial and temporal orientation and verbal and nonverbal communication.

**Table 2:** Description of the EGP items

ITEM	Function	Task/s	Further investigation
Static coordination I (M)	Stability in standing position	Standing for 5 s (from without support, to with one support, …, till impossible)	
Static coordination II (M)	Balance in different positions (unipedal stand, bipedal tiptoe, unipedal tip-toe)	Standing on your toes Standing on one foot Stand on the tip of a foot	
Dynamic coordination I (M)	Quality of walking	Walking (alone, with a stick, with a walker, …, till impossible)	
Dynamic coordination II (M)	Fast walking - Running over a short distance	Fast walking (10 m, 5 m) Slow running (10 m, 5 m)	
Joint mobilisation of upper limbs (Ph)	Joint Mobilisation of upper limbs	Passive mobilisation (wrist, elbow, shoulder) Active movements (wrist, elbow, shoulder)	Ask for information about the autonomy in dressing
Joint mobilisation of lower limbs (Ph)	Passive joint mobilisation of lower limbs (ankle, knee and hip)	Passive mobilization (ankle, knee, hip) Active movements (ankle, knee, hip)	
Hand fine motor skills (C)	Fingers dexterity	Buttoning and unbuttoning Finger tapping Finger-thumb opposition, Picking a coin	Ask about a possible loss of sensitivity at the fingertips Assess the participant’s capacity to denominate and attribute the right value to the coin
Lower extremity fine motor skills (M)	Eye-foot coordination	Foot placement on a footprint Kicking a ball Pointing with the foot	After the task, ask with which foot he thinks he hit first
Praxis (C)	Ideational apraxia Ideomotor apraxia	Use of cutlery Gestures of greeting, scolding, using of toothbrush, and nailing Writing Drawing on copy (circle, triangle; square, diagonal, median)	Ask the names of cutlery
	Constructional apraxia	6 cube-pyramid building (on model)	
Body representation (C)	Body part knowledge	On a model: identification of a person (front/back) On a model: identification of missing body parts Identification of body parts on verbal command Naming of body parts Posture imitation Immediate recall of posture	
Vigilance (C)	Sustained attention	Clinical evaluation at the end of the battery administration	
	Command execution	Grasping a cube on command (count to 5 and count to 10)	
		Identification of shapes and colours (circle, square and triangle, yellow, purple, red and green)	
Perceptual Memory (C)	Perceptual memory	Recall of previously seen colours Recall of body positions	
		Cued recall	
		Recognition	
Space (C)	Geographical location and orientation	Building, city	
	Knowledge of basic spatial concepts	Front/back, top/bottom concepts	
	Estimation of measures	Line bisection (10 cm and 15 cm)	
	Left/right orientation	Reciprocal positions of objects	
		Movement orientation	
Verbal Memory (C)	Semantic memory	Word immediate recall	
		5 min delayed recall	
	Episodic memory	Delayed recall	
		Cued recall	
		Recognition	
	Musical memory	Recall of “Happy birthday”	
Perception (C)	Auditory perception	Reproduction of rhythmic structures	
	Stereognosis	Tactile recognition (teaspoon, tennis ball)	
	Visual perception	Picture identification (cat, grape, mountain landscape)	
		Reading	
Temporal orientation (C)		Knowledge of days, months, current and birth dates	
		Reading time	
		Reordering and description of the sequence of events	
Communication (C)		Language consistency	
		Comprehension of instructions	
		Adequacy of nonverbal communication	
		Face expressivity	

For each item, the related component is reported in parentheses (C: cognitive Prevalence; M: motor Prevalence; ph: physical Constraints)

The French/Italian back-translation method was used to prepare the Italian version. Regarding the order of presentation of the items, the indications provided in the original manual were respected. The original scoring procedure was applied [28]. Each item was scored on a six-point scale (0–6) (see SM 2 for some examples). Some items had a set of sub-items (Table 2).

### Procedure

After the MMSE, the EGP was administered. Socio-demographical information, MNA-SF and GDS were collected before or after the assessment, depending on the circumstances (i.e. the presence of the caregiver) and the participants’ preferences.

The EGP was administered by a psychomotor therapist (SG) or psychologist (BM, AS, FE, BS, MV) previously trained on the administration and scoring procedure according to ethical principles and instructions, which remained consistent throughout the entire data collection. The examiners were not blinded to the patients’ diagnoses during EGP administration, as they were recruited and tested within clinical settings. For a subgroup of 29 healthy participants (8.71%), joint observations were collected, and the scores assigned by the two examiners were compared to reach shared parameters of judgment and calculate inter-rater reliability (SM 6). Moreover, internal consistency and Test-retest reliability were investigated (SM 6).

The assessment took on average from 50 to 70 min. The participants were allowed to take a break whenever they felt tired, in pain or uncomfortable [28, 43]. All participants (or their legal representative) gave their written informed consent, and the research was conducted in accordance with the guidelines of the Declaration of Helsinki (2013) and approved by the Local Ethical Committee (Prot. 29270, Prog. 926CESC).

### Statistical analyses

A preliminary analysis was conducted to identify any differences between the AD groups and healthy subjects regarding demographic and clinical variables.

Two separate series of analyses were carried out to investigate the patterns of ageing relating to AD and healthy ageing. At first, the four groups of AD patients were compared both to a group of age-matched healthy subjects (the 75-79- and 80–84 year old subgroups) and to each other. Furthermore, in a parallel analysis, the performances of the 7 HC groups (i.e. 60–64; 65–69; 70–74; 75–79; 80–84; 85–90; >90 years) were compared (SM 4). Given the variability intrinsically related to healthy and even more so to pathological ageing, the analyses were conducted on the entire samples, without removing outliers.

Both series of analyses were performed on the three EGP components’ scores identified by previous studies [29–31], namely *Cognitive prevalence*,* Motor prevalence*, and *Physical Constraints*.

The rationale behind investigating these three factors within the Italian context also relied on a clinically relevant reason related to the overall underestimation of physical and motor components, particularly when addressing the screening and treatment of AD patients. While in public health campaigns promoted to prevent the ageing-associated risks, motor activity and physical well-being are highly encouraged and recommended as crucial factors for maintaining individual health, diagnostic processes and interventions for dementia and AD usually tend to focus exclusively on the cognitive domain.

Moreover, for the sake of completeness, single-item analyses were also carried out for each component. According to the non-normal distribution of the data (Cognitive Prevalence: W = 0.83, *p* < 0.001; Motor prevalence: W = 0.66, *p* < 0.001; Physical Constraints: W = 0.89, *p* < 0.001), non-parametric Kruskal-Wallis Rank Sum Tests were used. Post-hoc comparisons were performed using the Pairwise Wilcoxon Rank Sum test, Bonferroni corrected (*afex* package of R statistical software, version 4.0.2). Finally, Spearman’s Correlations and linear regression analyses were performed to investigate any potential association between the EGP components and clinical and demographic variables.

## Results

### Preliminary examination

5 AD patients, > 90 years old, were excluded since a comparison with age-matched controls was impossible as they belonged to different AD severity groups (MMSE score: < 15 = 1; 15-19.50 = 2; 19.51–24.50 = 2). The clinical and demographic data for the remaining 89 AD patients and the 132 age-matched HCs are reported in Table 1 and SM 3.

### Psychomotor decline in Alzheimer’s Disease

#### Cognitive prevalence

**Fig. 1:**
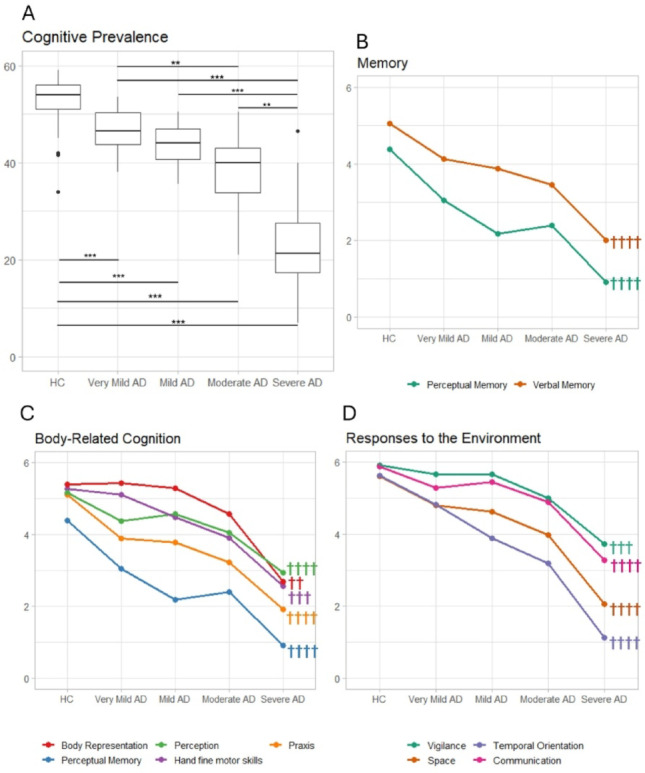
**A**. Boxplots of the Cognitive Prevalence score of the AD groups and HC showing the significant differences between the AD patients and HC and among the patients’ subgroups (significance levels: *p<0.05; **p<0.01; ***p<0.001). **B**, **C** and **D**: descriptive line plots of the performance of the AD and HC groups in the single items of the EGP Cognitive Prevalence Component aggregated into the Memory. Body-related Cognition and Responses to the Environment subdomains (symbol legend †††† differences since Very Mild AD in comparison to HC, ††† differences since Mild AD in comparison to HC; †† differences since Moderate AD in comparison to HC; † differences since Severe AD in comparison to HC)

For this component, a significant main effect of group (*H*_*(4)*_ = 140.28, *p* < 0.001) emerged. According to post-hoc tests, all the AD groups performed significantly worse than the HCs (*p* < 0.001, Fig. 1A). In addition, significant differences emerged when comparisons between the AD groups were made, in particular between Severe vs. Very Mild, Mild and Moderate AD (*p <* 0.001) and between Moderate vs. Very Mild (*p* = 0.004) (Table 3).

#### Comparisons between AD groups and HCs: single item analysis

All the AD groups performed worse than the controls in the Perceptual and Verbal Memory tasks (Table 3; Fig. 1B). For the body-related cognition tasks (Fig. 1C), all the AD groups failed in Perception and Praxis. Finally, in the environment-associated tasks (Fig. 1D), all the AD groups failed in Space Representation, Temporal Orientation, and Communication.

Moreover, all the AD groups except the Very Mild one performed worse than HC in the Hand fine motor skills test and Vigilance (i.e. sustained attention and command execution).

As far as Body representation is concerned, the differences become significant in the comparison between Moderate AD and the controls and, obviously, persist in the comparisons between Severe AD and the controls.

#### Comparisons between the four AD groups: single item analysis

There were significant differences between the Mild and Very Mild AD groups in temporal orientation, indicating a precocious decline of this function since a very early stage of the disease. This decline persists in the comparison between the Moderate and Very Mild AD groups, which also revealed the presence of differences in Hand Fine Motor skills.

The Severe AD group performed worse with respect to Very Mild, Mild and Moderate AD patients in Perceptual and Verbal Memory, Body Representation, Praxis, Space Representation, and Temporal Orientation (Table 3). Moreover, the performance of the Severe AD group was significantly impaired with respect to the Very Mild and Mild AD groups in the Hand Fine Motor Skills, Vigilance, Perception and Communication subtests.

**Table 3:** Post-hoc comparisons using Wilcoxon rank sum test Holm-Bonferroni corrected on the main effect of *Group* for the three EGP components and the related items: cognitive prevalence component (A), motor prevalence component (B), physical constraints component (C).and for the single items related to the component (B)

	Very Mild AD	Mild AD	Moderate AD	Severe AD
***A***				
***Cognitive Prevalence***				
Mild AD	0.5359	--	--	--
Moderate AD	**0.0041**	0.0675	--	--
Severe AD	**< 0.001**	**< 0.001**	**0.0012**	--
HC	**< 0.001**	**< 0.001**	**< 0.001**	**< 0.001**
***Hand fine motor skills***				
Mild AD	0.2273	--	--	--
Moderate AD	**0.0038**	0.7913	--	--
Severe AD	**0.0015**	**0.0076**	0.1256	--
HC	1.0000	**0.0003**	**< 0.001**	**< 0.001**
***Praxis***				
Mild AD	1.0000	--	--	--
Moderate AD	0.6764	0.7012	--	--
Severe AD	**0.0041**	**0.0009**	**0.0168**	--
HC	**0.0002**	**< 0.001**	**< 0.001**	**< 0.001**
***Body Representation***				
Mild AD	1.0000	--	--	--
Moderate AD	0.1023	0.1790	--	--
Severe AD	**< 0.001**	**< 0.001**	**0.0064**	--
HC	1.0000	1.0000	**0.0050**	**< 0.001**
***Vigilance***				
Mild AD	1.0000	--	--	--
Moderate AD	0.4490	0.7597	--	--
Severe AD	**0.0008**	**0.0001**	**0.0844**	--
HC	1.0000	**0.0183**	**< 0.001**	**< 0.001**
**Perceptual Memory**				
Mild AD	0.0656	--	--	--
Moderate AD	0.4108	1.0000	--	--
Severe AD	**< 0.001**	**0.0009**	**0.0019**	--
HC	**0.0001**	**< 0.001**	**< 0.001**	**< 0.001**
***Space***				
Mild AD	1.0000	--	--	--
Moderate AD	0.2170	0.6382	--	--
Severe AD	**0.0002**	**< 0.001**	**0.0011**	--
HC	**0.0140**	**< 0.001**	**< 0.001**	**< 0.001**
***Verbal Memory***				
Mild AD	1.0000	--	--	--
Moderate AD	0.4745	1.0000	--	--
Severe AD	**0.0014**	**0.0043**	**0.0532**	--
HC	**0.0101**	**0.0002**	**< 0.001**	**< 0.001**
***Perception***				
Mild AD	1.0000	--	--	--
Moderate AD	1.0000	0.9283	--	--
Severe AD	**0.0530**	**0.0033**	0.1082	--
HC	**0.0231**	**0.0328**	**< 0.001**	**< 0.001**
***Temporal Orientation***				
Mild AD	**0.0039**	--	--	--
Moderate AD	**< 0.001**	0.2382	--	--
Severe AD	**< 0.001**	**< 0.001**	**0.0001**	--
HC	**< 0.001**	**< 0.001**	**< 0.001**	**< 0.001**
***Communication***				
Mild AD	1.0000	--	--	--
Moderate AD	1.0000	1.0000	--	--
Severe AD	**0.0198**	**0.0016**	**0.1086**	--
HC	**0.0003**	**0.0064**	**< 0.001**	**< 0.001**
***B***				
***Motor Prevalence***				
Mild AD	1.0000	--	--	--
Moderate AD	1.0000	1.0000	--	--
Severe AD	0.4052	0.0759	1.0000	--
HC	1.0000	0.9839	**0.0204**	**0.0002**
***Static Coordination II***				
Mild AD	1.0000	--	--	--
Moderate AD	0.3960	0.9314	--	--
Severe AD	**0.0311**	0.0195	0.9354	--
HC	1.00000	0.6863	**0.0063**	**< 0.001**
***Dynamic Coordination I***				
Mild AD	1.0000	--	--	--
Moderate AD	1.0000	1.0000	--	--
Severe AD	1.0000	1.0000	1.0000	--
HC	**0.0123**	1.0000	**0.0038**	1.0000
***Dynamic coordination II***				
Mild AD	1.000	--	--	--
Moderate AD	1.000	1.000	--	--
Severe AD	1.000	1.000	1.000	--
HC	0.075	1.000	1.0000	**0.016**
***Lower extremity fine motor skills***				
Mild AD	0.9873	--	--	--
Moderate AD	0.6692	0.6692	--	--
Severe AD	0.0566	**0.0233**	0.6692	--
HC	0.6692	0.6692	**0.0019**	**< 0.001**
**C**				
***Physical Constraints***				
Mild AD	1.00000	--	--	--
Moderate AD	1.00000	1.00000	--	--
Severe AD	1.00000	1.00000	1.00000	--
HC	**0.0066**	**< 0.001**	**0.0013**	**0.0003**
***Joint mobilization of upper limbs***				
Mild AD	1.0000	--	--	--
Moderate AD	1.0000	1.00000	--	--
Severe AD	0.7133	0.9941	1.00000	--
HC	**0.046**	**0.0002**	**0.0067**	**0.0008**
***Joint mobilization of lower limbs***				
Mild AD	1.0000	--	--	--
Moderate AD	1.0000	1.00000	--	--
Severe AD	1.0000	1.0000	1.00000	--
HC	**0.0035**	**< 0.001**	**0.0098**	**0.0004**

Significant comparisons are reported in bold

#### Motor prevalence

**Fig. 2:**
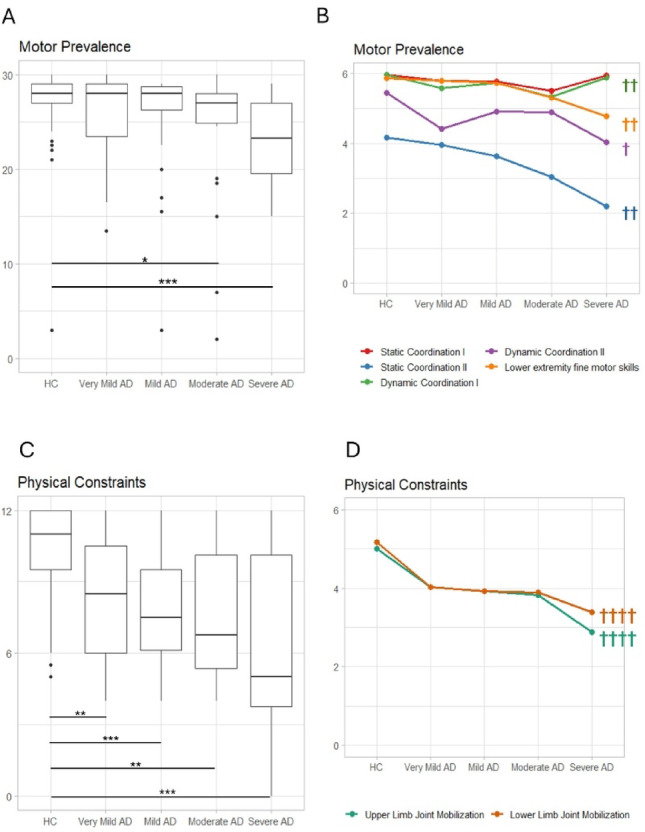
**A**. Boxplots of the Motor Prevalence score of the AD groups and HC showing the significant differences between the AD patients and HC and among the patients’ subgroups. **B**. Descriptive line plot of the AD and HC groups’ performance in the single items of the EGP Motor Prevalence Component. **C**. Boxplots of the Physical Constraints score of the AD groups and HC showing the significant differences between the AD patients and HC and among the patients’ subgroups. **D**. Descriptive line plot of the AD and HC groups’ performance in the single items of the EGP Physical Constraints Component. Significance levels: *p<0.05; **p<0.01; ***p<0.001. Symbol legend †††† differences since Very Mild AD in comparison to HC, ††† differences since Mild AD in comparison to HC; †† differences since Moderate AD in comparison to HC; † differences since Severe AD in comparison to HC

Significant differences between the groups emerged for *Motor prevalence* (*H*_(4)_ = 25.39, *p* < 0.001) (Fig. 2A). Post-hoc analyses showed a difference with respect to the controls for the Moderate (*p* = 0.02) and Severe AD (*p* < 0.001) groups, while no differences between the four AD groups were found. The same pattern of results with respect to the HCs also emerged in the single-item analysis (Table 3; Fig. 2B) except for Static Coordination I (i.e. standing for 5 s, also with a support). Furthermore, the Severe AD group performed worse than the Very Mild and Mild AD groups in Lower extremity fine motor skills and Static Coordination.

#### Physical constraints

For the *Physical Constraints* component and the items related to joint mobility in both upper and lower limbs, significant differences were found between the AD groups and HC (all comparisons: *p* < 0.01), while no differences between the AD groups emerged (Table 3; Fig. 2C and D).

### Patterns of cognitive and motor healthy ageing

#### Cognitive component

**Fig. 3:**
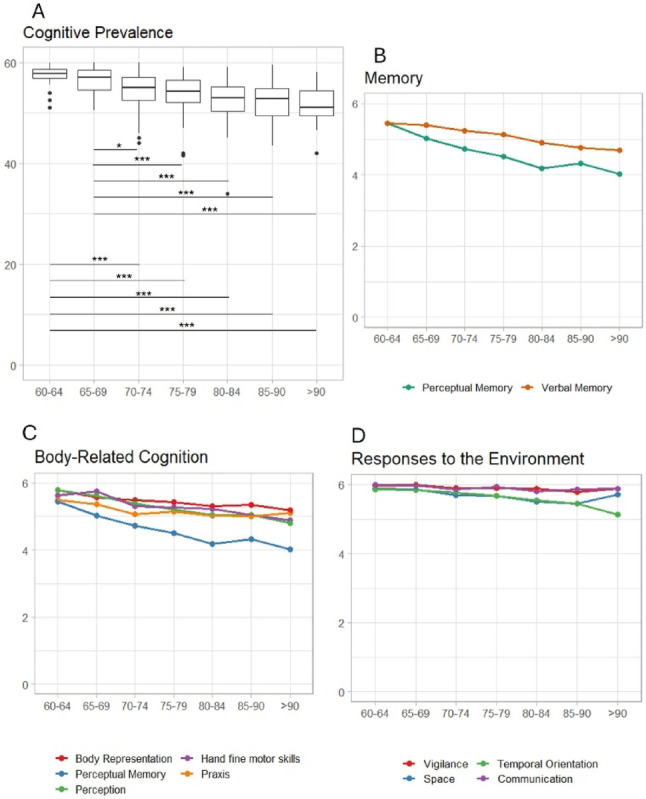
**A**. Boxplots of the Cognitive Prevalence score of the HC groups showing the significant differences between the seven subgroups. (significance levels: *p<0.05; **p<0.01; ***p<0.001). **B**, **C** and **D**: descriptive line plot of the performance of HC groups performance in the single items of the EGP Cognitive Prevalence Component aggregated into the Memory. Body-related Cognition and Responses to the Environment subdomains

A general cognitive decline due to ageing emerged in the *Cognitive component* (*H*_(6)_ = 78.10, *p* < 0.001), which becomes evident, in particular, in the transition between 65 and 69 and 70–74 years (Fig. 3A).

As far as the single-item analysis is concerned, the transition between the sixties and seventies was associated with a decline in Hand fine motor skills, Body representation, Perceptual Memory and Perception. For Verbal memory (Fig. 3C), significant differences were only found in the comparison between the 65–69 vs. 85–89 age groups (Fig. 3B), for Space Representation between 65 and 69 vs. 80–84 and for Temporal orientation between 65 and 69 vs. 80–84, and 60–64 vs. 85–89 (Fig. 3D). No significant differences between the groups emerged for Praxis, Vigilance and Communication (more details in Fig. 3 and SM 4.2.1).

#### Motor component and physical constraints

**Fig. 4:**
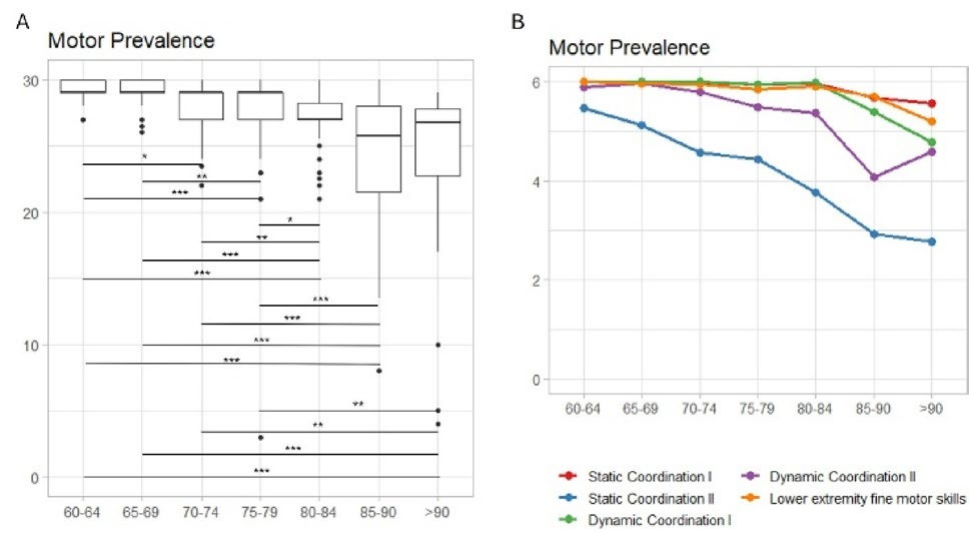
**A**. Boxplots of the Motor Prevalence score of the HC groups showing the significant differences between the seven HC subgroups. (significance levels: *p<0.05; **p<0.01; ***p<0.001). B. Descriptive line plot of the HC groups’ performance in the single items of the EGP Motor Prevalence Component

Regarding the *Motor component* (*H*_(6)_ = 99.58, *p* < 0.001), the decline seems to be gradual, becoming statistically significant after the seventies with respect to the sixties (Fig. 4A). This pattern was observed in particular for Static Coordination II (i.e. standing on one foot or on the toes), which moreover showed a second significant decline after 80 (Fig. 4B). After the eighties, the differences in motor performance become significant with respect to the younger groups, also for Dynamic coordination abilities (Fig. 4B).

Lower extremity fine motor skills seem to resist better, since a decline tends to manifest in particular after 90 (when the performance becomes different with respect to all the other groups except for the 85–89 group). Details are reported in Fig. 4 and SM 4.2.2.

Finally, no significant differences due to ageing were found in the *Physical constraint* component and its items (SM 4.2.3).

Due to space constraints, more details on the analyses performed on HCs are reported in SM 4.

### Associations of psycho-motor decline with other clinical measures and age in Alzheimer’s Disease

Spearman’s correlations were carried out to investigate potential associations between the EGP domains, as well as between these and the clinical measures collected (SM 5). A strong, positive correlation between the three EGP domains emerged in the AD sample, indicating that the performance in the motor domain was positively associated with the scores for the Cognitive Prevalence *(ρ* = 0.61*)* and Physical Constraints components *(ρ* = 0.53*)*, which, in turn, positively correlated with each other (*ρ* = 0.61*)* (Table SM5.1-A). Both the motor and cognitive components were positively associated with MMSE (Motor Prevalence: *ρ* ***=*** 0.39; Cognitive Prevalence: *ρ* ***=*** 0.68) and negatively with impairment in ADL (Motor Prevalence: *ρ* ***= − 0***.45; Cognitive Prevalence: *ρ* ***=*** -0.42). Similarly, the scores for the Physical Constraints component (i.e. hypertonia) were associated with greater impairment in daily life activities (ADL: *ρ* = -0.43; IADL: *ρ* = -0.32), while normal muscular tone was associated with better nutrition (MNA-SF: *ρ* = 0.38*)*.

Similar results were found for the age-matched HC and the total HC sample, which are reported in SM5 (Table SM5.1-B and SM5.2).

Moreover, linear regression analyses were performed to investigate the impact of MMSE and age on the EGP components (SM 5.3). The results showed that both the variables impact on the cognitive component when considering the total sample and the AD group (R^2^ ≥ 0.7), while for HC and the other components, R^2^ values of the linear regressions performed, although significant, are low (R^2^ < 0.21).

## Discussion

The study aimed to investigate early clinical signs and the progression of symptoms in AD patients by means of a comprehensive screening tool for cognitive, motor functions and muscle tone. Furthermore, the patterns of ageing in healthy people were analysed. The main results indicate that from the very early stages of AD (i.e. when symptoms are still in the initial phase, and the traditional screening used in clinical practice does not detect the presence of disorders, MMSE ≥ 24.51), specific tasks, involving not only the cognitive domain but also the motor and physical ones, can detect early initial symptoms.

Despite the predominant role of the cognitive component, as demonstrated by the fact that the gap between AD and HC tends to manifest earlier in the cognitive than in the motor domain, the strong correlation that emerged in the AD sample between the EGP Cognitive, Motor and Physical Constraints components seems to support the idea that the panorama of symptoms characterising AD extends beyond cognitive areas. Further support for this result derives from the association found between the Physical Constraints component and the general cognitive functioning in patients, which underscores the impact of the pathology in these less expected and explored domains.

Neither mood disturbances nor nutritional status (MNA-SF) are associated with performance in Cognitive and Motor Prevalence domains, while, as expected, EGP scores correlate with both MMSE and ADL in the AD group. Conversely, nutrition is associated with Physical Constraints items (i.e. alterations in muscle tone) in the AD group. In the control group, the Cognitive Prevalence score correlates with the MMSE and the motor prevalence score with the IADL.

From the perspective of an embodied approach to cognition, it is not surprising that a pathology such as AD (which has traditionally been considered only in terms of cognitive decline) actually also manifests in the body with altered functionality and representations [23]. Nevertheless, unfortunately, the role of the body in maintaining autonomy and well-being is still underestimated in clinical and neuropsychological interventions [44], thus explaining why, especially in clinical practice, the prevention and care of dementia still focus almost exclusively on pharmacological therapy and cognitive stimulation [45]. Taken together, these data suggest that advanced cognitive stimulation programs able to take into account all three domains investigated in the current study, namely cognitive functions, motor abilities and physical constraints, are needed [46].

### Potential early markers of AD

Although the EGP does not aim to provide an in-depth, function-specific assessment, its administration revealed some interesting differences in the patterns of psychomotor decline between AD patients and healthy controls, which seem to emerge since the earliest stages of the disease and may possibly represent early markers of the pathology.

In particular, within the *cognitive domain*, even at very mild and mild stages of AD (when autonomy in daily life is mostly spared), the patients’ performance was worse than the age-matched controls, not only, as expected, in memory-related domains (i.e. Perceptual and Verbal memory), Temporal orientation, Space Representation, Perceptive functions, Praxis, and Communication, but also in Fine motricity of the upper limbs. These differences emerged despite the fact that an initial decrease in cognitive performance due to physiological ageing had already occurred in the HCs (i.e. between the sixties and the seventies). Communication impairment emerged in our sample since the earliest stages of AD as well. This finding is in contrast with previous literature, according to which the decline in communicative abilities becomes evident only in the progression to moderate stages of the disease, regardless of sociodemographic variables [47, 48]. However, this discrepancy may be due to the fact that the standard of education in our sample was relatively low.

Another very early, perhaps unexpected symptom found in our AD sample regards *physical constraints*. A significant resistance to joint mobilisation (i.e. hypertonia, an increase in muscle tone in response to passive movement, which is proportional to the amount of force applied, [49]) in both lower and upper limbs during both active and passive movements emerged in the AD group, since the very mild stage of the illness. In EGP, this aspect is clinically tested by the examiner who rates the perceived resistance to passive mobilisation of upper and lower limbs, as well as the decreased joint range of motion during active movements. In our sample, these symptoms were not attributed to arthritic joint degeneration; however, other clinical comorbidities, such as diabetes mellitus or subclinical extrapyramidal features, can not be ruled out. Although hypertonia is also described in healthy ageing [50], it is usually more frequently reported in severe dementia, with a prevalence varying from 58% [51] to 100% [52]. Intriguingly, our data seem to be in line with the hypothesis that the alterations in muscle tone could represent a prodromal sign of dementia [51, 53], possibly due to impaired inhibition of motor responses resulting from damage to the frontal networks [53, 54] or unmasking of primordial infantile responses [52]. More recently, research has shown that peripheral biomechanical changes (e.g. accumulation of advanced glycation end-products -AGEs), resulting in intramuscular inflammation and skeletal muscle dysfunction, contribute to hypertonia in the early stages of dementia [55]. Despite the impact of hypertonia on motor functions, the associated discomfort and decreased quality of life, this condition is often underestimated among clinicians and researchers, and little is known about potential interventions to prevent and treat this symptom [56].

### Cognitive decline in AD

Besides the early manifestations described above, other changes occur later during the disease course. This is the case of abilities related to Body representation, which seem to resist pathological decline better in our AD sample: significant differences with respect to the controls were only found in the Moderate and Severe AD groups. A possible interpretation of this result could be related to the decline observed in our HC sample in the transition between the sixties and the seventies in body-related functions, rather than to the maintenance of this function in patients. However, the preservation of individual forms of body memory, even in the later stages of the illness, has been documented [57], in particular for: (1) *procedural memory*, at least for well-known and automatised actions and motor sequences; (2) *individual habits* (or *incorporative memory,* [58]), that is, “attitudes and roles that have often been taken over from others and have been incorporated as an embodied personality structure” [57] p. 671]; (3) *situational memory*, allowing patients to recognise familiar situations, environments and atmospheres and (4) *inter-corporeality*, the mimetic and gestural expressions that allow social interactions. These bodily memories probably help AD patients to maintain a certain degree of autonomy in routine daily life activities due to their familiarity. Moreover, the link between bodily memories and autonomy in daily life activities might explain why AD patients, when removed from their familiar environment, typically appear completely disoriented and unable to complete even the simplest actions. When clinically assessed, outside the context of everyday life, both praxis and hand fine motor skills seem to be compromised from the early stages of the illness.

In the Very Mild stage of AD, the absence of differences with respect to the controls within the vigilance domain is in contrast with previous studies, which showed a reduction in attention and executive functions from the earliest stages of AD and also in Mild Cognitive Impairment [59, 60]. It is worth noting that in the EGP, the Vigilance score is not assessed by means of specific tasks but derives from a clinical evaluation of the patients’ sustained attention throughout the tests and their ability to execute commands. In addition, the EGP does not require sustained attention over a long period of time, as the items are very short, and the alternation of different types of tasks probably helps to maintain the participants’ attention. Although during the EGP the examiner can take note of executive disorders such as perseverations, lack of response inhibition or reduced monitoring, for a comprehensive screening, the test should be associated with a more specific assessment of executive functions.

### Motor decline in AD

The results show that in Moderate stages of AD, motor decline becomes evident in terms of Balance (i.e. the ability to maintain a vertical position while standing still under different conditions, e.g., one-legged, or on one foot – Static coordination II), walking ability and eye-foot coordination (i.e. the ability to place a foot on a footprint, point with a foot or kick a ball – Dynamic coordination). This seems to confirm previous findings indicating a decline in functional mobility and balance in dementia patients, along with reduced manual dexterity [61]. Gate dysfunctions occur in over 30% of individuals suffering from MCI [62] and this proportion is likely to be greater in AD patients. These motor dysfunctions have been found to be associated with both medial temporal lobe degeneration and vessel disease [61] and they represent well-documented, non-secondary symptoms of AD. Furthermore, motor disorders increase the risk of falls and injuries, thus negatively affecting patients’ residual autonomy.

### Psychomotor ageing in healthy older adults

The patterns of cognitive ageing in healthy populations reveal that a first overall decline in cognitive performance can be detected in the transition between the ages of sixty and seventy. This mainly involves the cognitive functions which are more closely associated with sensory-motor functions such as Fine motor skills of the upper limbs, while a decline in Body Representation, Perceptual Memory and Perception seems to occur around the age of 75. Other functions, such as Vigilance, Temporal orientation and Space representation, decline much later, and this impairment tends to manifest around the age of eighty in comparison to younger groups. Communication, Verbal memory and Praxis abilities do not seem to be affected in our HC sample.

As far as the motor and muscle tone domain is concerned, we found that, overall, a significant decline tends to manifest from 75 years onwards, becoming more noticeable around 80. In particular, this decline appears to predominantly affect the ability to maintain balance in different positions (i.e. static coordination II) and walking (i.e. dynamic coordination), while eye-foot coordination (i.e. lower extremity fine motor skills) and joint mobility (i.e. Physical Constraints component) seem to resist better. In our sample, we only found a significant reduction in lower extremity fine motor skills in the older group (> 90), and no alterations in joint mobilisation due to ageing were recorded.

### Clinical implications

Beyond providing relevant clinical information on the patterns associated with psychomotor decline in both healthy and pathological ageing, our results demonstrate that the use of comprehensive batteries, such as the EGP, may provide relevant contributions to routine clinical practice and should be implemented for several reasons.

Firstly, the EGP is able not only to differentiate between AD patients and age-matched healthy subjects, but also to discriminate between the varying degrees of disease severity along three clinical dimensions (i.e. cognitive, motor and physical dimensions). Moreover, the battery enables clinicians to follow individual patterns of ageing in the geriatric, neurologically healthy population. This is facilitated by the fact that EGP offers the possibility of obtaining important clinical information about patients’ general and cognitive functioning, as well as about the perceptual, motor and physical abilities/functions, using a single instrument and in a relatively short time. It is indeed worth noting that the overall EGP administration time is relatively short, especially if compared to the administration time required to investigate each domain separately (i.e., using different tests for each domain). Related to this, another relevant aspect is the feasibility of the instrument, demonstrated by the fact that the structure of the EGP battery, with its simple, almost playful tasks and daily life activities involved, makes the tests pleasant and stress-free for the patients.

Moreover, the possibility of identifying early (not solely cognitive) markers of AD will improve not only the diagnostic process but also the treatment approach and strategies, which should include interventions specifically addressing motor functions and muscle tone.

## Limitations and future directions

There are some limitations in the study. Although the sample is large enough to compare the AD and HC groups, the AD subgroups differ in size, thereby increasing internal variability. The same limitation also concerns the HC sample. For this reason, we decided not to remove potential outliers from the statistical analyses to avoid a further reduction in the number of participants in each group. Therefore, further research on larger samples is needed to confirm the current results.

Another potential limitation may be represented by the fact that a comprehensive validation of the battery was not fully carried out. The number of joint observations and follow-up administrations is small, meaning that it was not possible to assess the inter-rater and test-retest reliability of the battery in a more consistent manner (see SM6 for details). However, it is noteworthy that the EGP was not originally devised with the aim of offering specific cut-off scores [28], but rather to provide a good preliminary clinical screening to identify potential deficits deserving further, ad hoc investigation. For this reason, we believe that the implementation of this tool could be most effective within the healthcare pathway, especially in primary care settings, during routine screening of the general older population, or within geriatric units.

Finally, it is well-known that social dimensions impact on the quality of ageing and unfortunately, no specific measures were collected in the current study. Considering all the aforementioned aspects, further studies are needed to provide a robust validation of the battery and to investigate more in-depth the role of other ageing-related aspects and dimensions, such as social dimensions, in order to explore their impact on the quality and patterns of both healthy and pathological ageing.

## Conclusions

The results of this study show that dementia (AD) does not exclusively affect cognitive functions. Crucially, it extends to bodily dimensions and the three domains involved (cognitive abilities, motricity and physical constraints) correlate with each other. This means that it is fundamental to consider the necessity of updating clinical and therapeutic approaches to ageing and mental deterioration to implement effective prevention protocols and to improve the quality of care. With respect to healthy ageing, our results show that body-related cognitive functions decline precociously, suggesting that specific prevention programs might contribute to the improvement of healthy lifespan in old age. In conclusion, our data suggest that psychomotor batteries, embracing a body-brain integrated approach to mental decline, may represent very useful tools for providing a comprehensive screening of the early clinical symptoms of AD and patterns of healthy ageing, as well as for implementing innovative and effective treatment strategies and prevention programmes. Although a more specific in-depth assessment of the deficits emerging from the administration of the EGP is necessary, the battery provides a comprehensive preliminary picture of an individual’s psychomotor abilities, and represents a valuable tool for repeated assessments, capable of detecting the emergence of any signs of decline precociously.

## Supplementary Information

Below is the link to the electronic supplementary material.

Supplementary Material 1

## Data Availability

The data that support the findings of this study are openly available in OSF at https://osf.io/y2rxv/?view_only=a458850271814d45b0d57f313b632d9a.
