# Supplementary material for: Patterns of cognitive and motor decline in Alzheimer’s Disease (AD) and ageing in healthy populations
Source: Aging Clin Exp Res. 2026 Jan 31;38(1):74. doi: 10.1007/s40520-026-03327-1 (PMC12886200; doi:10.1007/s40520-026-03327-1)
Supplement: Supplementary file 1 — Supplementary Material 1 [file 40520_2026_3327_MOESM1_ESM.docx]

**Patterns of cognitive and motor decline in Alzheimer’s Disease (AD) and ageing in healthy populations**

# SM1 - HC Sample Demographic and clinical characteristics

333 neurologically healthy subjects participated in the study. They were enrolled according to the same criteria used for the AD patients. The same demographic and clinical information (i.e. MMS, ADL and IADL, MNA-SF, GDS) collected on the AD patient sample was also collected for the healthy participants. The entire HC sample was considered for the analyses of the trajectories of psychomotor decline in healthy ageing. For this purpose, the sample was divided into seven age groups: 60-64, 65-69, 70-74, 75-79, 80-84, 85-90, and >90 years. The demographic and clinical data for the groups are reported in Table SM1 below.

|  | **HC 60-64** | **HC 65-69** | **HC 70-74** | **HC 75-79** | **HC 80-84** | **HC 85-89** | **HC >90** |
| --- | --- | --- | --- | --- | --- | --- | --- |
| **N** | 28 | 65 | 62 | 80 | 52 | 28 | 18 |
| **Female/Male** | 16/12 | 45/20 | 47/15 | 55/25 | 33/19 | 18/10 | 10/8 |
| **Age (y)** (M±SD) | 61.96 ± 1.62 | 66.98 ± 1.41 | 72.19 ± 1.48 | 77.2 ± 1.41 | 81.83 ± 1.42 | 86.57 ± 1.2 | 91.22 ± 1.66 |
| **Education** (M±SD) | 11.93 ± 2.99 | 10.58 ± 3.98 | 9.18 ± 4.11 | 8.2 ± 4.23 | 7.88 ± 4 | 6.64 ± 4.01 | 7.78 ± 4.45 |
| **Physically Active/non-active** | 20/8 | 50/12 | 49/12 | 54/21 | 33/19 | 14/14 | 6/11 |
| **MMSE**  (Folstein et al., 1975) | 28.97 ± 1.38 | 28.7 ± 1.39 | 28.83 ± 1.63 | 28.35 ± 1.63 | 28.13 ± 1.69 | 28.08 ± 1.58 | 28.1 ± 2.25 |
| **ADL ^$^** (mdn[range])  (Katz et al., 1963) | 0 [0] | 0 [0] | 0 [0] | 0 [0-1] | 0 [0-3] | 0 [0-2] | 0 [0-3] |
| **iADL ^$^** (mdn[range])  (Lawton & Brody, 1969) | 0 [0] | 0 [0] | 0 [0-1] | 0 [0-4] | 0 [0-2] | 0 [0-5] | 0.5 [0-4] |
| **MNA-SF ^$^** (M±SD)  (Rubenstein et al., 2001) | 12.83 ± 1.62 | 12.94 ± 1.29 | 12.49 ± 1.93 | 12.03 ± 2.26 | 11.66 ± 2.79 | 11.48 ± 3.14 | 10.71 ± 2.87 |
| **GDS ^$^** (M±SD)  (Yesavage et al., 1982) | 1.82 ± 1.49 | 2.02 ± 2.01 | 2.23 ± 2.06 | 2.19 ± 2.02 | 2.75 ± 2.44 | 4.07 ± 2.65 | 4.44 ± 2.83 |

Table SM1: Demographic characteristics of the HC groups clustered for age. MMSE: Mini-Mental State Examination; ADL: Activity of Daily Living (number of lost abilities); IADL: Instrumental Activity of Daily Living (number of lost abilities); MNA-SF: Mini Nutritional Assessment-Short Form (cut-off ≤10); GDS: Geriatric Depression Scale (cut-off ≥ 6). $ = missing data: ADL (0.3%), IADL (1.8%), GDS (3%), MNA-SF (17.7%).

# SM2 – EGP Scoring

Each of the EGP items is scored on a 0–6-point scale according to the instructions. Some examples of the scoring procedure are reported in the table below (Table SM2).

| **Item** | **Function** | **Description** | **Scoring** | **Total score** |
| --- | --- | --- | --- | --- |
| **Dynamic Coordination I (M)** | Quality of walking | Stand and walk for 5 meters back and forth | 6 points if the patient walks alone without help | Total  6 points |
|  |  |  | 5 points if the patient walks with a cane |  |
|  |  |  | 4 points if the patient walks using a walker |  |
|  |  |  | 3 points if the patient walks with the help of another person |  |
|  |  |  | 2 points if the patient can walk using a bilateral handrail |  |
|  |  |  | 1 point if the patient can walk with the help of two persons |  |
|  |  |  | 0 points if the patient is not able to walk. |  |
| **Joint mobilisation of upper limbs (Ph)** | Joint Mobilisation of upper limbs | passive mobilisation (all bilateral):  wrist, elbow, shoulder | 0.5 points for each passive mobilisation executed | Total  6 points |
|  |  |  | 0 points if the passive mobilisation is not possible |  |
|  |  | active movements (all bilateral):  wrist, elbow, shoulder | 0.5 points for each movement correctly executed |  |
|  |  |  | 0 points if the patient cannot reproduce any of the movements correctly |  |
| **Praxis (C)** | Ideational apraxia Ideomotor apraxia | Use of cutlery | 1 point if the patient can correctly use the cutlery | Total  6 points |
|  |  |  | 0 points if the patient is not able to use the cutlery/uses the cutlery in the wrong way. |  |
|  |  | Gestures (pantomime):  greeting, scolding, using a toothbrush, nailing | 0.5 points for each gesture correctly reproduced |  |
|  |  |  | 0 points if the patient is not able to reproduce any gesture. |  |
|  |  | Writing:  name, surname and date of birth | 1 point if the patient writes the name, surname and date of birth correctly |  |
|  |  |  | 0 points if the patient is not able to write, refuses to write, or the writing is incorrect |  |
|  |  | Drawing on a copy:  Fig1: circle+ triangle;  Fig 2: square, diagonal, median | 0.5 points for each figure correctly reproduced |  |
|  |  |  | 0 points if the patient cannot reproduce any of the figures correctly |  |
|  | Constructional Apraxia | 6 cube-pyramid building (on model) | 1 point if the patient can reproduce the model correctly in 15 seconds or less |  |
|  |  |  | 0.5 points if the patient can reproduce the model correctly within 16-30 seconds or reproduce the model in 15 seconds or less, but the spatial organisation is not completely correct (e.g. reduced or absent space between the cubes) |  |
|  |  |  | 0 points if the patient cannot reproduce the model or if the execution of the task takes more than 30 seconds. |  |

Table SM2: Description of the EGP items. For each item, the related component is reported in parentheses (C: Cognitive Prevalence; M: Motor Prevalence; Ph: Physical Constraints).

# SM3- Psychomotor Decline in Alzheimer’s Disease

## Preliminary Assessment, descriptive statistics and clinical characteristics: AD and Age-matched healthy controls

To investigate the presence of differences between the AD groups and the age-matched HCs (HC 75-79 and HC 84-84 groups), a series of preliminary analyses were conducted on the demographic and clinical data (Tables SM3.1 and SM3.2). The AD groups did not differ for age (H(4) = 7.72, p = 0.10), proportion of males to females (χ2(4) = 3.18, p = 0.53), or standard of education (H(4) = 5.99, p = 0.2). There was a difference between the groups in terms of the number of people who were physically active when the tests were carried out (i.e motor activity at least twice a week) (χ2(4) = 34.73, p < 0.001). In the Very Mild AD (p = 0.006) and Moderate AD (p < 0.001) groups the number of physically active patients was significantly lower than the HC group. 12.36% of the AD participants lived alone (36.36% in the matched HC), while 51.68% lived with a spouse (47.73% of the matched controls) and 13.48% with their family (12.88% of the matched controls). As expected, significant differences in the MMSE scores emerged (H(4) = 159.32, p < 0.001) between the AD groups (all comparisons: p < 0.001), reflecting the differing degrees of AD severity, as well as with respect to the controls (all comparisons p < 0.001). A similar pattern also emerged in terms of autonomy in daily life activities as measured by the ADL ( H(4) = 79.23, p < 0.001): impairment was greater in the Severe AD subgroup as compared to all the other AD groups (vs Very Mild AD: p = 0.01; vs Mild AD: p= 0.005; vs Moderate AD: p = 0.02), and all the patients (Very Mild, Mild, Moderate and Severe AD) had significantly lower scores than the controls (Very Mild AD vs HC: p = 0.004; Mild, Moderate, Severe AD vs HC: p < 0.001). There were also significant differences between AD patients and the HCs in the IADL scores (H(4) = 96.64, p < 0.001), demonstrating significant impairments in all of the AD groups with respect to the matched HCs (all comparisons: p < 0.001).

There were no significant differences relating to nutritional state, either between the AD groups or between the patients and controls (H(4) = 9.21, p = 0.06), although in AD, the risk of possible malnutrition (MNA-SF ≤ 10) is higher than in healthy people (AD 14/41, 34.15%, HC 28/114, 24.56%). The analysis performed on the GDS (H (4) = 9.67, p = 0.05) revealed that the Mild AD group showed mood alterations only when compared to the HCs (p = 0.05). The summary of the analyses performed is reported in the tables below (Table SM3.1). Post-hoc comparisons performed to investigate the differences between the groups (HC, Very Mild, Mild, Moderate, and Severe AD) are reported in Tables SM3.2.

|  | Statistic | P value |
| --- | --- | --- |
| Gender | Χ^2^ (4) = 3.183 | 0.528 |
| Age | H(4) = 7.722 | 0.102 |
| Education | H(4) = 5.998 | 0.199 |
| Physical Activity | Χ^2^ (4) = 34.733 | **<.0001** |
| Mini Mental State Examination (MMSE) | H(4) = 159.32 | **<.0001** |
| Activities of Daily Living (ADL) | H(4) = 79.232 | **<.0001** |
| Instrumental Activities of Daily Living (IADL) | H(4) = 96.637 | **<.0001** |
| Mini Nutritional Assessment-Short Form (MNA-SF) | H(4) = 9.206 | 0.056 |
| Geriatric Depression Scale (GDS) | H(4) = 9.667 | **0.046** |

Table SM3.1: Summary of the results of the analysis performed on the clinical and demographical data of the AD groups and age-matched HC sample. Chi-square and Kruskal-Wallis Rank Sum Test were used according to the data typology.

| ***Physical Activity*** | | Very Mild AD | | Mild AD | | Moderate AD | | Severe AD | |
| --- | --- | --- | --- | --- | --- | --- | --- | --- | --- |
| Mild AD | 0.684 | | -- | | -- | | -- | |  |
| Moderate AD | 1.000 | | 0.115 | | -- | | -- | |  |
| Severe AD | 1.000 | | 1.000 | | 0.715 | | -- | |  |
| HC | **0.006** | | 0.684 | | **<0.001** | | 0.434 | |  |
| ***Mini Mental State Examination (MMSE)*** | Very Mild AD | | Mild AD | | Moderate AD | | Severe AD | |  |
| Mild AD | **<0.001** | | -- | | -- | | -- | |  |
| Moderate AD | **<0.001** | | **<0.001** | | -- | | -- | |  |
| Severe AD | **<0.001** | | **<0.001** | | **<0.001** | | -- | |  |
| HC | **<0.001** | | **<0.001** | | **<0.001** | | **<0.001** | |  |
| ***Activities of Daily Living (ADL)*** | Very Mild AD | | Mild AD | | Moderate AD | | Severe AD | |  |
| Mild AD | 1.000 | | -- | | -- | | -- | |  |
| Moderate AD | 1.000 | | 1.000 | | -- | | -- | |  |
| Severe AD | **0.010** | | **0.005** | | **0.017** | | -- | |  |
| HC | **0.004** | | **<0.001** | | **<0.001** | | **<0.001** | |  |
| ***Instrumental Activities of Daily Living (IADL)*** | Very Mild AD | | Mild AD | | Moderate AD | | Severe AD | |  |
| Mild AD | 1.00 | | -- | | -- | | -- | |  |
| Moderate AD | 0.58 | | 0.65 | | -- | | -- | |  |
| Severe AD | 0.68 | | 0.86 | | 1.00 | | -- | |  |
| HC | **<0.001** | | **<0.001** | | **<0.001** | | **<0.001** | |  |
| ***Geriatric Depression Scale (GDS)*** | Very Mild AD | | Mild AD | | Moderate AD | | Severe AD | |  |
| Mild AD | 1.00 | | -- | | -- | | -- | |  |
| Moderate AD | 1.00 | | 1.00 | | -- | | -- | |  |
| Severe AD | 1.00 | | 1.00 | | 1.00 | | -- | |  |
| HC | 1.00 | | **0.05** | | 1.00 | | 1.00 | |  |

Table SM3.2: Post-hoc comparisons on the main effect of *Group on* clinical variables performed using Pairwise comparisons for χ^2^ test (Physical Activity) and Wilcoxon rank sum test Holm-Bonferroni corrected (MMSE score, Activities of Daily Living -ADL, Instrumental Activities of Daily Living -IADL (scores calculated as the number of lost abilities), and Geriatric Depression Scale -GDS).

## EGP scores: Statistical Analysis of AD groups and Age-matched healthy controls

In the table below (Table SM3.3), the means and standard deviations of the EGP components and single-item scores for the AD groups and age-matched HCs are presented.

|  | **HC**  **M(SD)** | **Very Mild AD**  **M(SD)** | **Mild AD**  **M(SD)** | **Moderate AD M(SD)** | **Severe AD**  **M(SD)** |
| --- | --- | --- | --- | --- | --- |
| ***Cognitive prevalence (0-60)*** | 53.33 (3.87) | 46.53 (4.72) | 43.75 (4.16) | 38.62 (7.54) | 23.19 (10.98) |
| Hand Fine Motor Skills | 5.26 (0.75) | 5.11 (0.74) | 4.47 (0.96) | 3.9 (1.04) | 2.56 (1.91) |
| Praxis | 5.1 (0.76) | 3.89 (1.17) | 3.78 (1.06) | 3.23 (1.12) | 1.91 (1.7) |
| Body Representation | 5.39 (0.53) | 5.42 (0.56) | 5.28 (0.69) | 4.56 (1.15) | 2.69 (1.66) |
| Vigilance | 5.90 (0.27) | 5.66 (0.78) | 5.65 (0.54) | 5.00 (1.25) | 3.72 (1.57) |
| Perceptual Memory | 4.38 (1.07) | 3.05 (1.04) | 2.18 (0.96) | 2.4 (1.33) | 0.91 (0.76) |
| Space | 5.61 (0.65) | 4.79 (1.33) | 4.62 (1.03) | 3.98 (1.27) | 2.06 (1.09) |
| Verbal Memory | 5.05 (0.95) | 4.13 (1.19) | 3.87 (1.40) | 3.46 (1.28) | 2.00 (1.40) |
| Perception | 5.15 (0.81) | 4.37 (1.14) | 4.57 (1.05) | 4.04 (1.15) | 2.94 (1.39) |
| Temporal Orientation | 5.62 (0.54) | 4.82 (0.69) | 3.88 (0.85) | 3.19 (1.11) | 1.12 (0.97) |
| Communication | 5.88 (0.52) | 5.29 (1.12) | 5.45 (1.04) | 4.88 (1.62) | 3.28 (1.91) |
| ***Motor Prevalence (0-30)*** | 27.39 (2.96) | 25.53 (4.93) | 25.75 (5.51) | 24.06 (7.03) | 22.81 (4.55) |
| Static Coordination I | 5.95 (0.45) | 5.79 (0.92) | 5.77 (0.97) | 5.50 (1.56) | 5.94 (0.25) |
| Static Coordination II | 4.17 (1.22) | 3.95 (1.61) | 3.63 (1.38) | 3.04 (1.52) | 2.19 (1.52) |
| Dynamic Coordination I | 5.95 (0.44) | 5.58 (1.02) | 5.73 (1.14) | 5.33 (1.66) | 5.88 (0.50) |
| Dynamic Coordination II | 5.44 (1.29) | 4.42 (2.03) | 4.9 (2.12) | 4.88 (2.18) | 4.03 (2.37) |
| Lower Extremity Fine Motor Skills | 5.87 (0.57) | 5.79 (0.54) | 5.72 (0.94) | 5.31 (1.14) | 4.78 (1.38) |
| ***Physical Constraints (0-12)*** | 10.19 (2.03) | 8.05 (2.69) | 7.83 (2.22) | 7.73 (2.92) | 6.25 (3.88) |
| Joint Mobilisation of the Upper limbs | 5.01 (1.15) | 4.03 (1.49) | 3.92 (1.35) | 3.83 (1.63) | 2.88 (2.15) |
| Joint Mobilisation of the Lower limbs | 5.18 (1.12) | 4.03 (1.49) | 3.92 (1.18) | 3.90 (1.47) | 3.38 (1.88) |

Table SM3.3: Means and Standard deviations of the EGP components and single items for the age-matched HC group and the AD groups.

### *Cognitive Prevalence*

Significant differences between the groups emerged when considering the Cognitive Prevalence score as well as the scores of the single items related to it (Table SM 3.4 ).

|  | Statistic | p-value |
| --- | --- | --- |
| Cognitive Prevalence | H(4)= 140.28 | **<.0001** |
| Hand Fine Motor Skills | H(4)= 62.569 | ***<* .0001** |
| Praxis | H(4)= 91.425 | **<.0001** |
| Body Representation | H(4)= 46.275 | **<.0001** |
| Vigilance | H(4)= 65.216 | **<.0001** |
| Perceptual Memory | H(4)= 113.85 | ***<* .0001** |
| Space | H(4)= 97.285 | ***<* .0001** |
| Verbal Memory | H(4)= 72.041 | ***<* .0001** |
| Perception | H(4)= 51.141 | ***<* .0001** |
| Temporal Orientation | H(4)= 146.63 | ***<* .0001** |
| Communication | H(4)= 63.119 | ***<* .0001** |

Table SM3.4: Summary of the Kruskal-Wallis tests performed on the EGP Cognitive Prevalence component and the related items to investigate the presence of differences between the groups.

### *Motor Prevalence*

Significant differences between the groups emerged when considering the overall Motor Prevalence score as well as the scores of the single items related to the component, except for Static Coordination I (Table SM3.5).

|  | Statistic | p-value |
| --- | --- | --- |
| Motor Prevalence | H(4)= 25.39 | **<.0001** |
| Static Coordination I | H(4)= 5.6737 | 0.225 |
| Static Coordination II | H(4)= 30.472 | **<.0001** |
| Dynamic Coordination I | H(4)= 14.027 | **0.007** |
| Dynamic Coordination II | H(4)= 13.615 | **0.009** |
| Lower Extremity Fine Motor Skills | H(4)= 33.982 | **<.0001** |

Table SM3.5: Summary of the Kruskal-Wallis tests performed on the EGP Motor Prevalence component and the related items to investigate the presence of differences between the groups.

### *Physical Constraints*

Significant differences between the groups emerged when considering the overall Physical Constraints component score as well as the scores of the single items related to it (Table SM3.6).

|  | Statistic | p-value |
| --- | --- | --- |
| Physical Constraints | H(4)= 48.413 | **<.0001** |
| Joint Mobilisation of the upper limbs | H(4)= 37.631 | **<.0001** |
| Joint Mobilisation of the lower limbs | H(4)= 48.258 | **<.0001** |

Table SM3.6: Summary of the Kruskal-Wallis tests performed on the EGP Physical Constraints component and the related items to investigate the presence of differences between the group.

# SM4 - Patterns of cognitive and motor ageing in healthy people

## Preliminary Assessment, descriptive statistics and clinical characteristics: Healthy controls

In keeping with what was done in the principal analysis on the AD patients and age-matched HCs, the analyses were performed on the seven groups of healthy participants described above (60-64;65-69; 70-74; 75-79; 80-84; 85-90; >90 years old) to investigate the trajectories for psychomotor decline in healthy ageing. A preliminary analysis investigated differences between the HC groups concerning the demographic and clinical variables. Also in this case, the analyses were performed using χ^2^ for gender and physical activity, while Kruskal-Wallis Rank Sum Tests were used for the other variables (i.e. education, MMSE, ADL, IADL, MNA-SF, GDS) according to the non-normal distribution of the data (Table SM4.1). Significant differences were found in education (H_(6)_= 48.11, *p* <0.001*)*, showing that participants older than 70 years (i.e. 70-74, 75-79, 80-84, 85-89, <90 groups) reported an average lower standard of education compared to the younger subjects (i.e. 60-64 and 65-69) (Table SM4.2). As far as physical activity is concerned (χ^2^_(6)_ = 22.74, *p* < 0.001), the proportion of healthy participants who were physically active at the time of the assessment was significantly lower in the oldest group (>90) compared to the 65-69 and 70-74 age groups (*p* = 0.02) (Table SM4.2). Regarding the autonomy in daily life activities, the analysis performed on the ADL scale revealed that the oldest participants (>90) had greater impairment compared to the younger groups, i.e. 60-64 (p = 0.02), 65-69 and 70-74 (p < 0.001), and 75-79 (p = 0.03) (Table SM4.2). Likewise, the 85-89 group reported a reduced level of autonomy compared to the 65-69 and 70-74 age groups (p = 0.01), while no differences emerged when comparing the older age groups to each other (i.e. 80-84, 85-89, >90). As far as instrumental daily life autonomy (IADL) is concerned, the age-related decline seems to appear earlier, as indicated by the significant difference found between the 80-84 age group and younger groups, respectively 65-69 (p < 0.001) and 70-74 (p= 0.001) (Table SM4.2). Similar to ADL, the 85-89 and > 90 age groups showed impaired functional autonomy as compared to the younger ones (from 60-64 to 75-79), and, also in this case, no differences between the older groups emerged. The analysis conducted on mood-related aspects revealed that older participants (85-89 and > 90) reported a higher prevalence of mood disorders as compared to the younger ones (60-64, 65-69, 70-74, 75-79, all comparisons p < 0.05) (Table SM4.2). Concerning general cognitive functioning (MMSE) and nutritional state (MNA-SF), the post hoc analysis did not confirm the presence of differences between the groups (Table SM4.2). Details of the analysis and the post-hoc comparisons performed on the HC groups are reported in the tables below (Tables SM4.1 and SM4.2).

|  | Statistic | p-value |
| --- | --- | --- |
| Gender | Χ^2^ (6) = 5.13 | 0.53 |
| Physical Activity | Χ^2^ (6) = 22.74 | ***<* 0.001** |
| Education | H(6) = 48.11 | ***<* 0.001** |
| Mini Mental State Examination (MMSE) | H(6) = 14.95 | **0.02** |
| Activities of Daily Living (ADL) | H(6) = 39.02 | ***<* 0.001** |
| Instrumental Activities of Daily Living (IADL) | H(6) = 65.32 | ***<* 0.001** |
| Mini Nutritional Assessment -Short Form (MNA) | H(6) = 14.49 | **0.02** |
| Geriatric Depression Scale (GDS) | H(6) = 26.01 | **0.0002** |

Table SM4.1: Summary of the results of the analysis performed on the clinical and demographical data of the HC groups. Chi-square and Kruskal-Wallis Rank Sum Test were used according to the data typology.

|  |  |  | |  | |  | |  | |  | |
| --- | --- | --- | --- | --- | --- | --- | --- | --- | --- | --- | --- |
| ***Physical Activity*** | HC 60-64 | HC 65-69 | | HC 70-74 | | HC 75-79 | | HC 80-84 | | HC 85-89 | |
| HC 65-69 | 1.000 | -- | | -- | | -- | | -- | | -- | |
| HC 70-74 | 1.000 | 1.000 | | -- | | -- | | -- | | -- | |
| HC 75-79 | 1.000 | 1.000 | | 1.000 | | -- | | -- | | -- | |
| HC 80-84 | 1.000 | 0.936 | | 0.952 | | 1.000 | | -- | | -- | |
| HC 85-89 | 1.000 | 0.125 | | 0.136 | | 0.936 | | 1.000 | | -- | |
| HC >90 | 0.618 | **0.017** | | **0.019** | | 0.165 | | 0.957 | | 1.000 | |
| ***Education*** | HC 60-64 | | HC 65-69 | | HC 70-74 | | HC 75-79 | | HC 80-84 | HC 85-89 |  |
| HC 65-69 | 1.0000 | | -- | | -- | | -- | | -- | -- |  |
| HC 70-74 | **0.0292** | | 0.8572 | | -- | | -- | | -- | -- |  |
| HC 75-79 | **0.0001** | | **0.0034** | | 1.0000 | | -- | | -- | -- |  |
| HC 80-84 | **0.0005** | | **0.0124** | | 1.0000 | | 1.000 | | -- | -- |  |
| HC 85-89 | **<0.001** | | **0.0002** | | 0.0373 | | 0.5649 | | 1.000 | -- |  |
| HC >90 | **0.013** | | 0.0779 | | 1.0000 | | 1.0000 | | 1.0000 | 1.000 |  |
| ***Mini Mental State Examination (MMSE)*** | HC 60-64 | HC 65-69 | | HC 70-74 | | HC 75-79 | | HC 80-84 | | HC 85-89 | |
| HC 65-69 | 1.00 | -- | | -- | | -- | | -- | | -- | |
| HC 70-74 | 1.00 | 1.00 | | -- | | -- | | -- | | -- | |
| HC 75-79 | 1.00 | 1.00 | | 0.72 | | -- | | -- | | -- | |
| HC 80-84 | 0.63 | 1.00 | | 0.17 | | 1.00 | | -- | | -- | |
| HC 85-89 | 0.13 | 0.75 | | 0.13 | | 1.00 | | 1.00 | | -- | |
| HC >90 | 1.00 | 1.00 | | 1.00 | | 1.00 | | 1.00 | | 1.00 | |
| **Activities of Daily Living (ADL)** | HC 60-64 | HC 65-69 | | HC 70-74 | | HC 75-79 | | HC 80-84 | | HC 85-89 | |
| HC 65-69 | -- | -- | | -- | | -- | | -- | | -- | |
| HC 70-74 | -- | -- | | -- | | -- | | -- | | -- | |
| HC 75-79 | 1.000 | 0.433 | | 0.496 | | -- | | -- | | -- | |
| HC 80-84 | 1.000 | 0.202 | | 0.238 | | 1.000 | | -- | | -- | |
| HC 85-89 | 0.382 | **0.009** | | **0.012** | | 1.000 | | 1.000 | | -- | |
| HC >90 | **0.024** | **<0.001** | | **<0.001** | | **0.034** | | 0.278 | | 1.000 | |
| **Activities of Daily Living (ADL)** | HC 60-64 | HC 65-69 | | HC 70-74 | | HC 75-79 | | HC 80-84 | | HC 85-89 | |
| HC 65-69 | -- | -- | | -- | | -- | | -- | | -- | |
| HC 70-74 | -- | -- | | -- | | -- | | -- | | -- | |
| HC 75-79 | 1.000 | 0.433 | | 0.496 | | -- | | -- | | -- | |
| HC 80-84 | 1.000 | 0.202 | | 0.238 | | 1.000 | | -- | | -- | |
| HC 85-89 | 0.382 | **0.009** | | **0.012** | | 1.000 | | 1.000 | | -- | |
| HC >90 | **0.024** | **<0.001** | | **<0.001** | | **0.034** | | 0.278 | | 1.000 | |
| **Instrumental Activities of Daily Living (IADL)** | HC 60-64 | HC 65-69 | | HC 70-74 | | HC 75-79 | | HC 80-84 | | HC 85-89 | |
| HC 65-69 | -- | -- | | -- | | -- | | -- | | -- | |
| HC 70-74 | 1.000 | 1.000 | | -- | | -- | | -- | | -- | |
| HC 75-79 | 1.000 | 0.1647 | | 0.7728 | | -- | | -- | | -- | |
| HC 80-84 | 0.0567 | **0.0002** | | **0.0014** | | 0.2270 | | -- | | -- | |
| HC 85-89 | **0.0162** | **<0.001** | | **0.0017** | | **0.0438** | | 1.000 | | -- | |
| HC >90 | **0.0010** | **<0.001** | | **<0.001** | | **0.0014** | | 0.7002 | | 1.000 | |
| **Mini Nutritional Assessment-SF (MNA-SF)** | HC 60-64 | HC 65-69 | | HC 70-74 | | HC 75-79 | | HC 80-84 | | HC 85-89 | |
| HC 65-69 | 1.000 | -- | | -- | | -- | | -- | | -- | |
| HC 70-74 | 1.000 | 1.000 | | -- | | -- | | -- | | -- | |
| HC 75-79 | 1.000 | 1.000 | | 1.000 | | -- | | -- | | -- | |
| HC 80-84 | 1.000 | 0.695 | | 1.000 | | 1.000 | | -- | | -- | |
| HC 85-89 | 1.000 | 0.531 | | 1.000 | | 1.000 | | 1.000 | | -- | |
| HC >90 | 0.344 | 0.054 | | 0.323 | | 1.000 | | 1.000 | | 1.000 | |
| **Geriatric Depression Scale (GDS)** | HC 60-64 | HC 65-69 | | HC 70-74 | | HC 75-79 | | HC 80-84 | | HC 85-89 | |
| HC 65-69 | 1.000 | -- | | -- | | -- | | -- | | -- | |
| HC 70-74 | 1.000 | 1.000 | | -- | | -- | | -- | | -- | |
| HC 75-79 | 1.000 | 1.000 | | 1.000 | | -- | | -- | | -- | |
| HC 80-84 | 1.000 | 1.000 | | 1.000 | | 1.000 | | -- | | -- | |
| HC 85-89 | **0.023** | **0.011** | | **0.031** | | **0.026** | | 0.556 | | -- | |
| HC >90 | **0.032** | **0.019** | | **0.050** | | **0.035** | | 0.484 | | 1.000 | |

Table SM4.2: Post-hoc comparisons on the main effect of *Group on* clinical variables performed using Pairwise comparisons for χ^2^ test (Physical Activity) and Wilcoxon rank sum test Holm-Bonferroni corrected (MMSE score, Activities of Daily Living -ADL, Instrumental Activities of Daily Living -IADL (scores calculated as the number of lost abilities), and Geriatric Depression Scale -GDS).

## EGP Scores: Statistical Analysis on healthy subjects

In line with the analyses conducted on the AD patients, a first inspection of the data was carried out to check for data distribution. According to the non-normal distribution of the data (Cognitive Prevalence: W = 0.92, *p* < 0.001; Motor Prevalence: W= 0.59, *p* < 0.001; Physical Constraints: W = 0.80, *p* < 0.001), the analyses were performed using Kruskal-Wallis Rank sum Tests.

In the table below (Table SM4.3), the means and standard deviations of the EGP components and single-item scores for the HC groups are reported.

|  | **HC 60-64** | **HC 65-69** | **HC 70-74** | **HC 75-79** | **HC 80-84** | **HC 85-89** | **HC >90** |
| --- | --- | --- | --- | --- | --- | --- | --- |
| ***Cognitive prevalence (0-60)*** | 57.34 (2.24) | 56.42 (2.25) | 54.44 (3.61) | 53.91 (3.56) | 52.45 (4.2) | 52.09 (4.47) | 51.36 (3.95) |
| Hand Fine Motor Skills | 5.64 (0.58) | 5.75 (0.48) | 5.31 (0.71) | 5.27 (0.73) | 5.24 (0.78) | 5.05 (1.06) | 4.89 (1.04) |
| Praxis | 5.50 (0.56) | 5.37 (0.63) | 5.07 (0.78) | 5.15 (0.75) | 5.03 (0.78) | 5.00 (0.83) | 5.11 (0.85) |
| Body Representation | 5.79 (0.35) | 5.57 (0.39) | 5.49 (0.52) | 5.44 (0.47) | 5.31 (0.6) | 5.36 (0.61) | 5.19 (0.71) |
| Vigilance | 5.98 (0.09) | 5.99 (0.06) | 5.9 (0.36) | 5.91 (0.26) | 5.88 (0.27) | 5.79 (0.52) | 5.89 (0.21) |
| Perceptual Memory | 5.45 (0.72) | 5.03 (0.83) | 4.73 (1.15) | 4.51 (0.99) | 4.18 (1.17) | 4.32 (1.26) | 4.03 (1.22) |
| Space | 5.89 (0.25) | 5.87 (0.28) | 5.69 (0.47) | 5.67 (0.58) | 5.51 (0.75) | 5.46 (0.64) | 5.72 (0.43) |
| Verbal Memory | 5.45 (0.55) | 5.39 (0.75) | 5.23 (0.8) | 5.14 (0.84) | 4.91 (1.1) | 4.77 (0.93) | 4.69 (0.96) |
| Perception | 5.79 (0.42) | 5.62 (0.47) | 5.4 (0.7) | 5.22 (0.81) | 5.04 (0.82) | 5.04 (0.79) | 4.81 (0.62) |
| Temporal Orientation | 5.86 (0.3) | 5.85 (0.29) | 5.77 (0.38) | 5.68 (0.5) | 5.54 (0.58) | 5.45 (0.55) | 5.14 (0.78) |
| Communication | 6.00 (0.00) | 5.98 (0.14) | 5.87 (0.46) | 5.92 (0.38) | 5.81 (0.69) | 5.86 (0.45) | 5.89 (0.47) |
| ***Motor Prevalence (0-30)*** | 29.36 (0.73) | 29.00 (1.01) | 28.27 (1.71) | 27.66 (3.42) | 26.98 (2.00) | 23.77 (5.37) | 22.89 (8.21) |
| Static Coordination I | 6.00 (0.00) | 5.98 (0.12) | 5.98 (0.13) | 5.94 (0.56) | 5.96 (0.19) | 5.68 (0.67) | 5.56 (1.10) |
| Static Coordination II | 5.46 (0.58) | 5.11 (0.87) | 4.56 (1.18) | 4.44 (1.19) | 3.77 (1.17) | 2.93 (1.68) | 2.78 (1.59) |
| Dynamic Coordination I | 6.00 (0.00) | 6.00 (0.00) | 6.00 (0.00) | 5.94 (0.56) | 5.98 (0.14) | 5.39 (0.99) | 4.78 (1.99) |
| Dynamic Coordination II | 5.89 (0.57) | 5.95 (0.37) | 5.78 (0.76) | 5.49 (1.31) | 5.37 (1.27) | 4.07 (2.41) | 4.58 (2.55) |
| Lower Extremity Fine Motor Skills | 6.00 (0.00) | 5.95 (0.26) | 5.94 (0.29) | 5.85 (0.67) | 5.9 (0.36) | 5.7 (0.85) | 5.19 (1.65) |
| ***Physical Constraints (0-12)*** | 9.71 (2.47) | 9.59 (2.54) | 10.45 (2.26) | 10.09 (2.04) | 10.35 (2.03) | 9.70 (2.12) | 9.78 (2.24) |
| Joint Mobilization Upper limbs | 4.79 (1.38) | 4.68 (1.29) | 5.23 (1.19) | 4.93 (1.16) | 5.13 (1.13) | 4.80 (1.05) | 4.81 (1.25) |
| Joint Mobilization Lower limbs | 4.93 (1.38) | 4.91 (1.36) | 5.22 (1.18) | 5.16 (1.11) | 5.21 (1.14) | 4.89 (1.29) | 4.97 (1.14) |

Table SM4.3: Means and Standard deviations of the EGP components and single items for HC groups.

### *4.2.1. Cognitive Prevalence*

Significant differences between the groups emerged when considering both the general Cognitive Prevalence component and the single items included, except for Communication (Table SM4.4). A general cognitive decline due to ageing is shown in the results regarding the performance of healthy participants in the *Cognitive Prevalence component* (*H*_(6)_= 78.10, *p* < 0.001) when clustered for age (60-64;65-69; 70-74; 75-79; 80-84; 85-90; >90 years). In particular, this decline becomes evident in the transition between 65-69 and 70-74 years (Table SM4.5 and Figure SM4.1).

As far as the single-item analysis is concerned, the transition between the sixties and the seventies is associated with a decline for Hand fine motor skills (65-69 v. 70- 74, *p =* 0.001), Body representation (60-64 v. 75- 79, *p* < 0.01), Perceptual Memory (60-64 v.75- 9, p < 0.001; 65-69 vs 75-79, *p* = 0.02) and Perception (60-64 v. 75- 79, *p* = 0.001; 65-69 v. 75- 79, *p*  = 0.02). For Verbal memory, significant differences were found in the comparison between 65-69 v. 85-89 (*p* = 0.03), for Space Representation between 65-69 vs 80-84, *p* = 0.02 and for Temporal orientation between 65-69 v. 80-84 (*p* = 0.03) and 60-64 vs 85-89 (*p* = 0.02). Post-hoc analyses on Praxis, Vigilance, and Communication revealed no significant differences between the groups*.* Details of the analyses and post-hoc comparisons performed on the EGP Cognitive Prevalence component and the related items are reported in the table below (Tables SM4.5 and SM4.6).

|  | Statistic | p-value |
| --- | --- | --- |
| Cognitive Prevalence | H(6)= 78.105 | **<.0001** |
| Hand Fine Motor Skills | H(6)= 35.036 | ***<* .0001** |
| Praxis | H(6)= 15.187 | **0.019** |
| Body Representation | H(6)= 23.093 | **0.001** |
| Vigilance | H(6)= 13.634 | **0.034** |
| Perceptual Memory | H(6)= 42.457 | ***<* .0001** |
| Space | H(6)= 21.423 | **0.001** |
| Verbal Memory | H(6)= 19.549 | **0.003** |
| Perception | H(6)= 53.326 | ***<* .0001** |
| Temporal Orientation | H(6)= 39.257 | ***<* .0001** |
| Communication | H(6)= 6.5194 | 0.368 |

Table SM4.4: Summary of the Kruskal-Wallis tests performed on the EGP Cognitive Prevalence component and the related items to investigate the presence of differences between the HC groups.

| ***Cognitive Prevalence*** | HC 60-64 | HC 65-69 | HC 70-74 | HC 75-79 | HC 80-84 | HC 85-89 |
| --- | --- | --- | --- | --- | --- | --- |
| HC 65-69 | 1.00000 | -- | -- | -- | -- | -- |
| HC 70-74 | **0.00087** | **0.03361** | -- | -- | -- | -- |
| HC 75-79 | **<0.001** | **<0.001** | 1.00000 | -- | -- | -- |
| HC 80-84 | **<0.001** | **<0.001** | 0.06717 | 0.50383 | -- | -- |
| HC 85-89 | **<0.001** | **<0.001** | 0.22418 | 1.00000 | 1.00000 | -- |
| HC >90 | **<0.001** | **<0.001** | 0.07368 | 0.19049 | 1.00000 | 1.00000 |

Table SM4.5: Post-hoc comparisons using Wilcoxon rank sum test Holm-Bonferroni corrected on the main effect of *Group* for Cognitive Prevalence

component*.* Significant comparisons are reported in bold

| **Hand fine motor skills** | HC 60-64 | HC 65-69 | HC 70-74 | HC 75-79 | HC 80-84 | HC 85-89 |
| --- | --- | --- | --- | --- | --- | --- |
| HC 65-69 | 1.00000 | -- | -- | -- | -- | -- |
| HC 70-74 | 0.48647 | **0.00139** | -- | -- | -- | -- |
| HC 75-79 | 0.22489 | **0.00018** | 1.00000 | -- | -- | -- |
| HC 80-84 | 0.29496 | **0.00077** | 1.00000 | 1.00000 | -- | -- |
| HC 85-89 | 0.27139 | **0.00225** | 1.00000 | 1.00000 | 1.00000 | -- |
| HC >90 | 0.17193 | **0.00454** | 1.00000 | 1.00000 | 1.00000 | 1.00000 |
| ***Praxis*** | HC 60-64 | HC 65-69 | HC 70-74 | HC 75-79 | HC 80-84 | HC 85-89 |
| HC 65-69 | 1.000 | -- | -- | -- | -- | -- |
| HC 70-74 | 0.159 | 0.547 | -- | -- | -- | -- |
| HC 75-79 | 0.569 | 1.000 | 1.00000 | -- | -- | -- |
| HC 80-84 | 0.078 | 0.236 | 1.00000 | 1.00000 | -- | -- |
| HC 85-89 | 0.261 | 0.788 | 1.00000 | 1.00000 | 1.00000 | -- |
| HC >90 | 1.000 | 1.000 | 1.00000 | 1.00000 | 1.00000 | 1.00000 |

| ***Body Representation*** | HC 60-64 | HC 65-69 | HC 70-74 | HC 75-79 | HC 80-84 | HC 85-89 |
| --- | --- | --- | --- | --- | --- | --- |
| HC 65-69 | 0.2089 | -- | -- | -- | -- | -- |
| HC 70-74 | 0.1671 | 1.0000 | -- | -- | -- | -- |
| HC 75-79 | **0.0066** | 1.0000 | 1.00000 | -- | -- | -- |
| HC 80-84 | **0.0025** | 0.5313 | 1.00000 | 1.00000 | -- | -- |
| HC 85-89 | **0.0467** | 1.0000 | 1.00000 | 1.00000 | 1.00000 | -- |
| HC >90 | **0.0045** | 0.4718 | 1.00000 | 1.00000 | 1.00000 | 1.00000 |
| ***Vigilance*** | HC 60-64 | HC 65-69 | HC 70-74 | HC 75-79 | HC 80-84 | HC 85-89 |
| HC 65-69 | 1.000 | -- | -- | -- | -- | -- |
| HC 70-74 | 1.000 | 0.914 | -- | -- | -- | -- |
| HC 75-79 | 1.000 | 0.446 | 1.000 | -- | -- | -- |
| HC 80-84 | 1.000 | 0.052 | 1.000 | 1.000 | -- | -- |
| HC 85-89 | 1.000 | 0.067 | 1.000 | 1.000 | 1.000 | -- |
| HC >90 | 1.000 | **0.026** | 1.000 | 1.000 | 1.000 | 1.000 |
| ***Perceptual Memory*** | HC 60-64 | HC 65-69 | HC 70-74 | HC 75-79 | HC 80-84 | HC 85-89 |
| HC 65-69 | 0.3032 | -- | -- | -- | -- | -- |
| HC 70-74 | 0.0645 | 1.0000 | -- | -- | -- | -- |
| HC 75-79 | **0.0003** | **0.0171** | 1.0000 | -- | -- | -- |
| HC 80-84 | **<0.001** | **0.0011** | 0.1978 | 1.0000 | -- | -- |
| HC 85-89 | **0.0092** | 0.1351 | 1.0000 | 1.0000 | 1.0000 | -- |
| HC >90 | **0.0019** | **0.0408** | 0.5670 | 1.0000 | 1.0000 | 1.0000 |
| ***Space*** | HC 60-64 | HC 65-69 | HC 70-74 | HC 75-79 | HC 80-84 | HC 85-89 |
| HC 65-69 | 1.0000 | -- | -- | -- | -- | -- |
| HC 70-74 | 0.6417 | 0.2264 | -- | -- | -- | -- |
| HC 75-79 | 1.0000 | 0.4190 | 1.0000 | -- | -- | -- |
| HC 80-84 | 0.1255 | **0.0173** | 1.0000 | 1.0000 | -- | -- |
| HC 85-89 | 0.0604 | **0.0093** | 1.0000 | 1.0000 | 1.0000 | -- |
| HC >90 | 1.0000 | 1.0000 | 1.0000 | 1.0000 | 1.0000 | 1.0000 |
| ***Verbal Memory*** | HC 60-64 | HC 65-69 | HC 70-74 | HC 75-79 | HC 80-84 | HC 85-89 |
| HC 65-69 | 1.000 | -- | -- | -- | -- | -- |
| HC 70-74 | 1.000 | 1.000 | -- | -- | -- | -- |
| HC 75-79 | 1.000 | 0.984 | 1.000 | -- | -- | -- |
| HC 80-84 | 1.000 | 0.349 | 1.000 | 1.000 | -- | -- |
| HC 85-89 | 0.088 | **0.035** | 0.593 | 1.000 | 1.000 | -- |
| HC >90 | 0.119 | 0.065 | 0.696 | 1.000 | 1.000 | 1.000 |
| ***Perception*** | HC 60-64 | HC 65-69 | HC 70-74 | HC 75-79 | HC 80-84 | HC 85-89 |
| HC 65-69 | 1.0000 | -- | -- | -- | -- | -- |
| HC 70-74 | 0.0597 | 1.0000 | -- | -- | -- | -- |
| HC 75-79 | **0.0011** | **0.0182** | 1.0000 | -- | -- | -- |
| HC 80-84 | **< 0.001** | **0.0004** | 0.2200 | 1.0000 | -- | -- |
| HC 85-89 | **0.0002** | **0.0015** | 0.3355 | 1.0000 | 1.0000 | -- |
| HC >90 | **< 0.001** | **< 0.001** | **0.0177** | 0.1421 | 1.0000 | 1.0000 |
| ***Temporal Orientation*** | HC 60-64 | HC 65-69 | HC 70-74 | HC 75-79 | HC 80-84 | HC 85-89 |
| HC 65-69 | 1.0000 | -- | -- | -- | -- | -- |
| HC 70-74 | 1.0000 | 1.0000 | -- | -- | -- | -- |
| HC 75-79 | 1.0000 | 0.6504 | 1.0000 | -- | -- | -- |
| HC 80-84 | 0.2782 | **0.0333** | 0.7924 | 1.0000 | -- | -- |
| HC 85-89 | **0.0209** | **0.0010** | 0.0437 | 0.3376 | 1.0000 | -- |
| HC >90 | **0.0015** | **< 0.001** | **0.0015** | **0.0135** | 0.7283 | 1.0000 |

Table SM4.6: Post-hoc comparisons using Wilcoxon rank sum test Holm-Bonferroni corrected on the main effect of *Group* for each item of the Cognitive Component*.* Significant comparisons are reported in bold.

### *Motor Prevalence*

Significant differences between the groups emerged when considering both the motor component and the single items related to it (Table SM4.7). With regard to the *Motor Prevalence component* (*H*_(6)_= 99.58, *p*  < 0.001), the decline seems to be gradual, becoming statistically significant after the seventies with respect to the sixties (70-74 vs 60-64, *p* = 0.02). After 80, the differences in motor performance are significant with respect to all the other younger groups (Table SM4.8). This is particularly evident for both items for dynamic coordination. For Static Coordination II, a first decline is evident between the sixties and the seventies (60-64 vs 70-74, p < 0.01; 65-69 vs 75-79, *p* < 0.01) and later after eighty (75-79 vs 80-84, *p* = 0.02). Lower limb fine motor skills seem to resist ageing better since the decline tends to manifest in particular after the age of 90*.* Details on the analyses and post-hoc comparisons performed on the EGP Motor Prevalence component and the related items are reported in the tables below (Tables SM4.8 and SM4.9).

|  | Statistic | p-value |
| --- | --- | --- |
| Motor Prevalence | H(6)=99.579 | **<.0001** |
| Static Coordination I | H(6)=32.86 | **<.0001** |
| Static Coordination II | H(6)=97.578 | **<.0001** |
| Dynamic Coordination I | H(6)=40.151 | **<.0001** |
| Dynamic Coordination II | H(6)=40.151 | **<.0001** |
| Lower Extremity Fine Motor Skills | H(6)=28.366 | **<.0001** |

Table SM4.7: Summary of the Kruskal-Wallis tests performed on the EGP Motor Prevalence component and the related items to investigate the presence of differences between the HC groups.

| ***Motor Prevalence*** | HC 60-64 | HC 65-69 | HC 70-74 | HC 75-79 | HC 80-84 | HC 85-89 |
| --- | --- | --- | --- | --- | --- | --- |
| HC 65-69 | 1.0000 | -- | -- | -- | -- | -- |
| HC 70-74 | **0.0239** | 0.2898 | -- | -- | -- | -- |
| HC 75-79 | **0.0004** | **0.0046** | 1.0000 | -- | -- | -- |
| HC 80-84 | **< 0.001** | **< 0.001** | **0.0024** | **0.0147** | -- | -- |
| HC 85-89 | **< 0.001** | **< 0.001** | **< 0.001** | **< 0.001** | 0.1644 | -- |
| HC >90 | **< 0.001** | **< 0.001** | **0.0021** | **0.0069** | 1.0000 | 1.0000 |

Table SM4.8: Post-hoc comparisons using Wilcoxon rank sum test Holm-Bonferroni corrected on the main effect of *Group* for the Motor Prevalence component*.* Significant comparisons are reported in bold.

| ***Static coordination I*** | HC 60-64 | HC 65-69 | HC 70-74 | HC 75-79 | HC 80-84 | HC 85-89 |
| --- | --- | --- | --- | --- | --- | --- |
| HC 65-69 | 1.0000 | -- | -- | -- | -- | -- |
| HC 70-74 | 1.0000 | 1.0000 | -- | -- | -- | -- |
| HC 75-79 | 1.0000 | 1.0000 | 1.0000 | -- | -- | -- |
| HC 80-84 | 1.0000 | 1.0000 | 1.0000 | 1.0000 | -- | -- |
| HC 85-89 | 0.2264 | **0.0180** | **0.0244** | **0.0054** | 0.2396 | -- |
| HC >90 | 0.6105 | 0.1617 | 0.1997 | 0.0742 | 1.0000 | 1.0000 |
| ***Static coordination II*** | HC 60-64 | HC 65-69 | HC 70-74 | HC 75-79 | HC 80-84 | HC 85-89 |
| HC 65-69 | 1.0000 | -- | -- | -- | -- | -- |
| HC 70-74 | **0.0088** | 0.1910 | -- | -- | -- | -- |
| HC 75-79 | **0.0002** | **0.0048** | 1.0000 | -- | -- | -- |
| HC 80-84 | **<0.001** | **<0.001** | **0.0118** | **0.0162** | -- | -- |
| HC 85-89 | **<0.001** | **<0.001** | **0.0003** | **0.0002** | 1.0000 | -- |
| HC >90 | **<0.001** | **<0.001** | **0.0008** | **0.0008** | 0.4616 | 1.0000 |

| ***Dynamic coordination I*** | HC 60-64 | HC 65-69 | HC 70-74 | HC 75-79 | HC 80-84 | HC 85-89 |
| --- | --- | --- | --- | --- | --- | --- |
| HC 65-69 | -- | -- | -- | -- | -- | -- |
| HC 70-74 | -- | -- | -- | -- | -- | -- |
| HC 75-79 | 1.0000 | 1.0000 | 1.0000 | -- | -- | -- |
| HC 80-84 | 1.0000 | 1.0000 | 1.0000 | 1.0000 | -- | -- |
| HC 85-89 | **0.0109** | **<0.001** | **<0.001** | **<0.001** | **0.0005** | -- |
| HC >90 | **0.0238** | **<0.001** | **<0.001** | **<0.001** | **0.002** | 1.0000 |
| ***Dynamic coordination II*** | HC 60-64 | HC 65-69 | HC 70-74 | HC 75-79 | HC 80-84 | HC 85-89 |
| HC 65-69 | 1.0000 | -- | -- | -- | -- | -- |
| HC 70-74 | 1.0000 | 1.0000 | -- | -- | -- | -- |
| HC 75-79 | 1.0000 | 0.1041 | 1.0000 | -- | -- | -- |
| HC 80-84 | 0.7967 | **0.0117** | 0.8807 | 1.0000 | -- | -- |
| HC 85-89 | **0.0100** | **<0.001** | **0.0007** | **0.0179** | 0.2409 | -- |
| HC >90 | 0.3574 | **0.003** | 0.3867 | 1.0000 | 1.0000 | 1.0000 |
| ***Lower extremity fine motor skills*** | HC 60-64 | HC 65-69 | HC 70-74 | HC 75-79 | HC 80-84 | HC 85-89 |
| HC 65-69 | 1.0000 | -- | -- | -- | -- | -- |
| HC 70-74 | 1.0000 | 1.0000 | -- | -- | -- | -- |
| HC 75-79 | 1.0000 | 1.0000 | 1.0000 | -- | -- | -- |
| HC 80-84 | 1.0000 | 1.0000 | 1.0000 | 1.0000 | -- | -- |
| HC 85-89 | 0.4475 | 0.7170 | 1.0000 | 1.0000 | 1.0000 | -- |
| HC >90 | **0.0093** | **0.0015** | **0.0086** | **0.0103** | **0.0455** | 1.0000 |

Table SM4.9: Post-hoc comparisons using Wilcoxon rank sum test Holm-Bonferroni corrected on the main effect of *Group* for each item of the Motor Prevalence Component. Significant comparisons are reported in bold.

### Physical Constraints

No significant differences due to ageing were found in the *Physical constraint* component and its subtest (Table SM4.10 and Figure SM4.3).

|  | Statistic | p-value |
| --- | --- | --- |
| Physical Constraints | H(6)=7.035 | 0.318 |
| Joint Mobilisation of the upper limbs | H(6)=9.111 | 0.167 |
| Joint Mobilisation of the lower limbs | H(6)=3.737 | 0.712 |

Table SM4.10: Summary of the Kruskal-Wallis tests performed on the EGP Physical Constraints component and the related items to investigate the presence of differences between the HC groups.


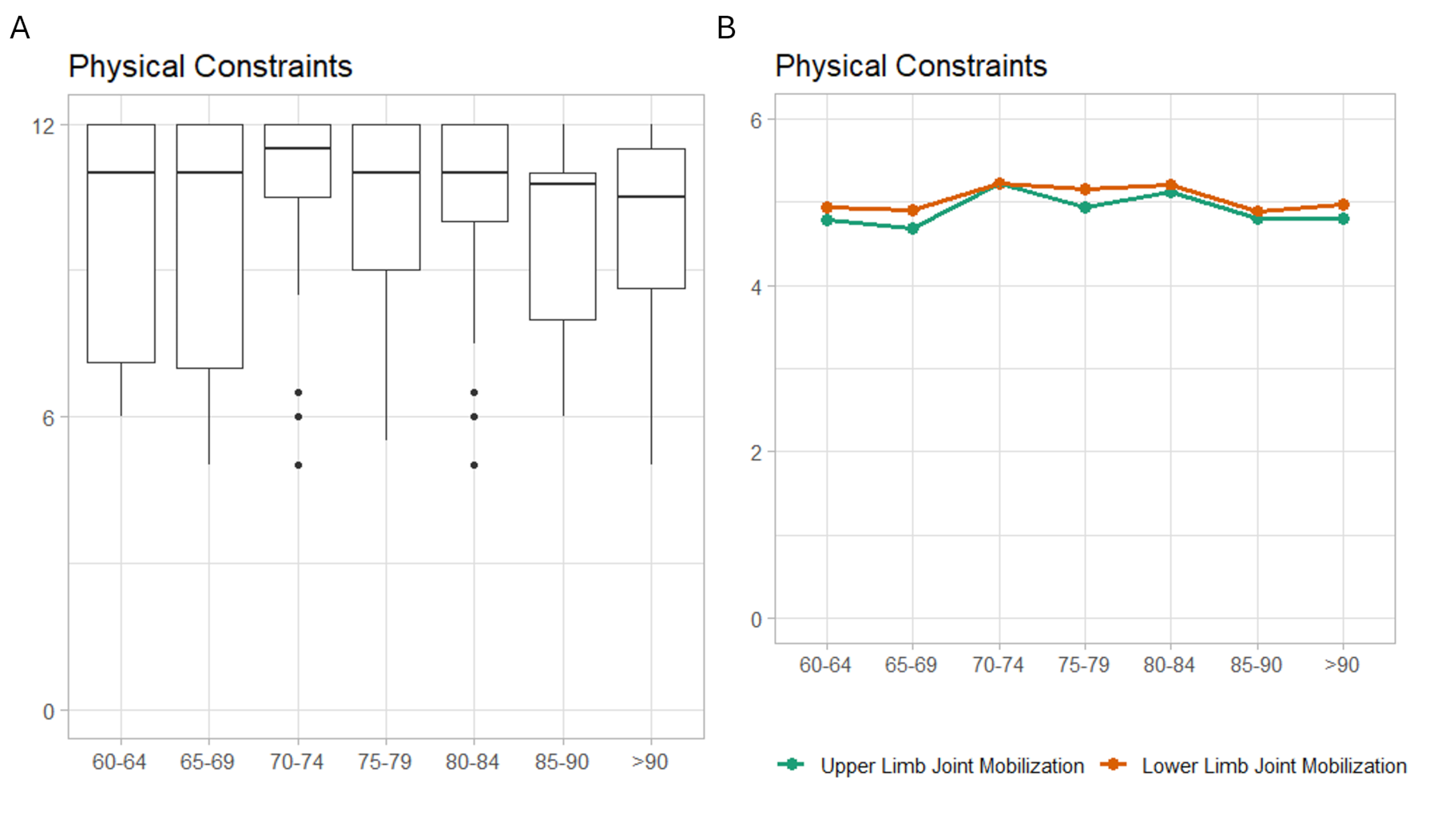


Figure SM4.3. A. Boxplots of the Physical Constraints score of the HCs showing the performance of the seven HC subgroups. B. Descriptive line plot of the HC groups’ performance in the single items of the EGP Physical Constraints Component.

# SM5 - Associations with other clinical measures in healthy people

## Correlation analyses on AD and Age-matched HC

In keeping with the correlation analyses performed on the AD sample, Spearman’s correlations were performed on the age-matched HC (n=132) (Table SM5.1). The results (Table SM5.1-B) showed a pattern similar to that observed in the AD sample (Table SM5.1-A), with a positive association between the Motor and Cognitive Prevalence domains (ρ= 0.35), and a trend towards significance for the correlation between Motor Prevalence and Physical Constraints Components (ρ= 0.27). With regard to the associations between the three EGP components and clinical measures, the motor component was negatively associated with the degree of impairment in daily life autonomy (ADL: ρ= -0.39), while the cognitive component correlated positively with the level of general cognitive functioning (MMSE: ρ= 0.33). Moreover, we found that the degree of impairment in instrumental daily life activities in the HC group correlated negatively with the nutritional condition (ρ= -0.31) and positively with mood as measured by the GDS (ρ= 0.36), meaning that when nutrition and mood are worse, impairment in daily life activities is greater.

| **A** |  |  |  |  |  |  |  |  |
| --- | --- | --- | --- | --- | --- | --- | --- | --- |
| ***AD sample*** | **Motor Prevalence** | **Cognitive Prevalence** | **Physical Constraints** | **MMSE** | **ADL** | **IADL** | **MNA-SF** | **GDS** |
| **Motor Prevalence** | 1.00 | -- | -- | -- | -- | -- | -- | -- |
| **Cognitive Prevalence** | **0.61** | 1.00 | -- | -- | -- | -- | -- | -- |
| **Physical Constraints** | **0.53** | **0.61** | 1.00 | -- | -- | -- | -- | -- |
| **MMSE**  (Folstein et al., 1975) | **0.39** | **0.68** | 0.24 | 1.00 | -- | -- | -- | -- |
| **ADL**  (Katz et al., 1963) | **-0.45** | **-0.42** | **-0.43** | -0.17 | 1.00 | **--** | -- | -- |
| **iADL**  (Lawton & Brody, 1969) | -0.20 | -0.25 | **-0.32** | -0.27 | **0.47** | 1.00 | -- | -- |
| **MNA-SF**  (Rubenstein et al., 2001) | 0.19 | 0.23 | **0.38** | 0.16 | -0.03 | 0.01 | 1.00 | -- |
| **GDS**  (Yesavage et al., 1982) | -0.25 | -0.04 | -0.10 | 0.25 | -0.01 | 0.13 | -0.12 | 1.00 |
| **B** |  |  |  |  |  |  |  |  |
| **HC sample** | **Motor Prevalence** | **Cognitive Prevalence** | **Physical Constraints** | **MMSE** | **ADL** | **IADL** | **MNA-SF** | **GDS** |
| **Motor Prevalence** | 1.00 | -- | -- | -- | -- | -- | -- | -- |
| **Cognitive Prevalence** | **0.35** | 1.00 | -- | -- | -- | -- | -- | -- |
| **Physical Constraints** | 0.11 | 0.27 | 1.00 | -- | -- | -- | -- | -- |
| **MMSE**  (Folstein et al., 1975) | 0.16 | **0.33** | 0.09 | 1.00 | -- | -- | -- | -- |
| **ADL**  (Katz et al., 1963) | -0.22 | -0.09 | -0.13 | -0.19 | 1.00 | -- | -- | -- |
| **iADL**  (Lawton & Brody, 1969) | **-0.39** | -0.28 | -0.09 | -0.20 | **0.36** | 1.00 | -- | -- |
| **MNA-SF**  (Rubenstein et al., 2001) | 0.13 | 0.12 | 0.09 | 0.17 | -0.18 | **-0.31** | 1.00 | -- |
| **GDS**  (Yesavage et al., 1982) | -0.17 | -0.19 | -0.05 | -0.16 | 0.09 | **0.36** | -0.19 | 1.00 |

Table SM5.1: **A.** Spearman’s correlations between EGP components and the clinical measures collected on the AD sample (n= 89). **B.** Spearman’s correlations between EGP components and the clinical measures collected on the AD-matched healthy controls’ sample (n= 132). Significant correlations are reported in bold. MMSE: Mini Mental State Examination; ADL: Activities of Daily Living (quantified as the number of activities for which the autonomy is lost); IADL: Instrumental Activities of Daily Living (quantified as the number of activities for which the autonomy is lost); MNA-SF: Mini Nutritional Assessment-Short Form; GDS: Geriatric Depression Scale

## Correlation analyses on the total HC sample (n =333)

The same analyses described in the previous section were also carried out on the entire HC sample (see Table SM5.2), confirming a positive, strong correlation between the Motor and Cognitive Prevalence components (ρ= 0.48). In keeping with the results on the subsample of AD-matched health subjects, the cognitive component was positively associated with the MMSE scores (ρ= 0.30). Moreover, both the components negatively correlated with the IADL (Motor Prevalence – IADL: ρ= -0.37; Cognitive Prevalence – IADL: ρ= -0.30), which was in turn associated with mood (i.e. GDS, ρ= 0.35).

|  | **Motor Prevalence** | **Cognitive Prevalence** | **Physical Constraints** | **MMSE** | **ADL** | **IADL** | **MNA-SF** | **GDS** |
| --- | --- | --- | --- | --- | --- | --- | --- | --- |
| **Motor Prevalence** | 1.00 | **0.48** | -- | -- | -- | -- | -- | -- |
| **Cognitive Prevalence** | **0.48** | 1.00 | -- | -- | -- | -- | -- | -- |
| **Physical Constraints** | 0.10 | 0.18 | 1.00 | -- | -- | -- | -- | -- |
| **MMSE**  (Folstein et al., 1975) | 0.25 | **0.30** | 0.03 | 1.00 | -- | -- | -- | -- |
| **ADL**  (Katz et al., 1963) | -0.25 | -0.15 | -0.04 | -0.15 | 1.00 | -- | -- | -- |
| **iADL**  (Lawton & Brody, 1969) | **-0.37** | **-0.30** | -0.11 | -0.15 | **0.33** | 1.00 | -- | -- |
| **MNA-SF**  (Rubenstein et al., 2001) | 0.25 | 0.21 | 0.11 | 0.08 | -0.18 | -0.27 | 1.00 | -- |
| **GDS**  (Yesavage et al., 1982) | -0.19 | -0.22 | -0.03 | -0.06 | 0.10 | **0.35** | -0.20 | 1.00 |

Table SM5.2: Spearman’s correlations between EGP components and the clinical measures collected for the entire healthy controls sample (n = 333). Significant correlations are reported in bold. MMSE: Mini Mental State Examination; ADL: Activities of Daily Living (quantified as the number of activities for which the autonomy is lost); IADL: Instrumental Activities of Daily Living (quantified as the number of activities for which the autonomy is lost); MNA-SF: Mini Nutritional Assessment-Short Form; GDS: Geriatric Depression Scale.

## Linear Regressions

The results of the linear regressions performed on the three EGP components ad MMSE, and Age are reported in the tables below (Tables SM5.3-SM5.5 and Figures SM5.1 and SM5.2).

| **Cognitive Prevalence (total sample)**  lm(Cognitive_Prevalence ~ MMSE + Age) | | | | | | |
| --- | --- | --- | --- | --- | --- | --- |
|  | Df | Sum Sq | Mean Sq | F | p |  |
| MMSE | 1 | 17266.511 | 17266.511 | 757.986 | <0.001 | *** |
| Age | 1 | 26.871 | 26.871 | 1.180 | 0.279 |  |
| Residuals | 218 | 4965.924 | 22.779 |  |  |  |
| R^2^: 0.78 | | | | | |  |
| **Cognitive Prevalence (HC sample)** | | | | | | |
|  | Df | Sum Sq | Mean Sq | F | p |  |
| MMSE | 1 | 245.329 | 245.329 | 20.161 | <0.001 | *** |
| Age | 1 | 149.785 | 149.785 | 12.309 | 0.001 | *** |
| Residuals | 129 | 1569.719 | 12.168 |  |  |  |
| R^2^: 0.20 | | | | | |  |
| **Cognitive Prevalence (AD sample)** | | | | | | |
|  | Df | Sum Sq | Mean Sq | F | p |  |
| MMSE | 1 | 6703.638 | 6703.638 | 197.507 | <0.001 | *** |
| Age | 1 | 149.459 | 149.459 | 4.403 | 0.039 | * |
| Residuals | 86 | 2918.949 | 33.941 |  |  |  |
| R^2^: 0.70 | | | | | |  |

Table SM5.3: Results of the linear regression performed on the EGP Cognitive Prevalence Component, with MMSE and Age. Df: degrees of freedom; Sum Sq: Sum of Squares; Mean Sq: mean square; F: value of the F statistic; p: p-value. Significance levels: ***: ≤.001; **<.01; *<.05); R^2^ : R-squared.

| **Motor Prevalence (total sample)**  lm(Motor_Prevalence ~ MMSE + Age) | | | | | | |
| --- | --- | --- | --- | --- | --- | --- |
|  | Df | Sum Sq | Mean Sq | F | p |  |
| MMSE | 1 | 477.962 | 477.962 | 27.984 | <0.001 | *** |
| Age | 1 | 200.479 | 200.479 | 11.738 | 0.001 | *** |
| Residuals | 218 | 3723.453 | 17.080 |  |  |  |
| R^2^: 0.15 | | | | | |  |
| **Motor Prevalence (HC sample)** | | | | | | |
|  | Df | Sum Sq | Mean Sq | F | p |  |
| MMSE | 1 | 94.436 | 94.436 | 11.758 | 0.001 | *** |
| Age | 1 | 13.686 | 13.686 | 1.704 | 0.194 |  |
| Residuals | 129 | 1036.036 | 8.031 |  |  |  |
| R^2^: 0.10 | | | | | |  |
| **Motor Prevalence (AD sample)** | | | | | | |
|  | Df | Sum Sq | Mean Sq | F | p |  |
| MMSE | 1 | 67.610 | 67.610 | 2.232 | 0.139 |  |
| Age | 1 | 205.703 | 205.703 | 6.791 | 0.011 | * |
| Residuals | 86 | 2605.164 | 30.293 |  |  |  |
| R^2^: 0.10 | | | | | |  |

Table SM5.4: Results of the linear regression performed on the EGP Motor Prevalence Component, with MMSE and Age. Df: degrees of freedom; Sum Sq: Sum of Squares; Mean Sq: mean square; F: value of the F statistic; p: p-value. Significance levels: ***: ≤.001; **<.01; *<.05); R^2^ : R-squared.

| **Physical Constraints (total sample)**  lm(Physical_Constraints ~ MMSE + Age) | | | | | | |
| --- | --- | --- | --- | --- | --- | --- |
|  | Df | Sum Sq | Mean Sq | F | p-value |  |
| MMSE | 1 | 324.326 | 324.326 | 54.556 | <0.001 | *** |
| Age | 1 | 15.621 | 15.621 | 2.628 | 0.106 |  |
| Residuals | 218 | 1295.981 | 5.945 |  |  |  |
| R^2^: 0.21 | | | | | |  |
| **Physical Constraints (HC sample)** | | | | | | |
|  | Df | Sum Sq | Mean Sq | F | p-value |  |
| MMSE | 1 | 0.004 | 0.004 | 0.001 | 0.975 |  |
| Age | 1 | 0.005 | 0.005 | 0.001 | 0.971 |  |
| Residuals | 129 | 539.314 | 4.181 |  |  |  |
| R^2^: 0.00001 | | | | | |  |
| **Physical Constraints (AD sample)** | | | | | | |
|  | Df | Sum Sq | Mean Sq | F | p-value |  |
| MMSE | 1 | 35.326 | 35.326 | 4.481 | 0.037 | * |
| Age | 1 | 16.861 | 16.861 | 2.139 | 0.147 |  |
| Residuals | 86 | 677.908 | 7.883 |  |  |  |
| R^2^: 0.07 | | | | | |  |

Table SM5.5: Results of the linear regression performed on the EGP Physical Constraints Component, with MMSE and Age. Df: degrees of freedom; Sum Sq: Sum of Squares; Mean Sq: mean square; F: value of the F statistic; p: p-value. Significance levels: ***: ≤.001; **<.01; *<.05); R^2^ : R-square


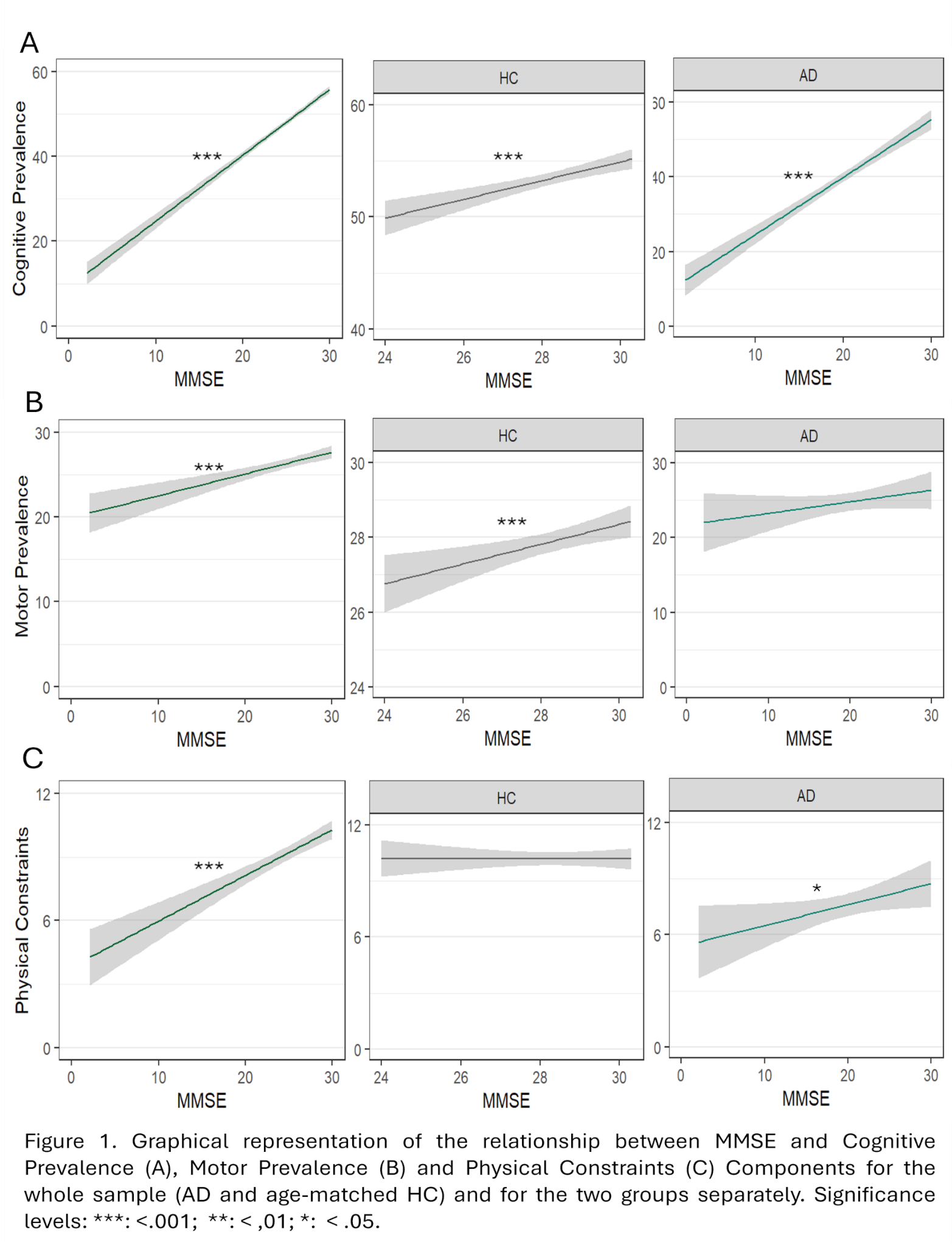


Figure SM5.1. Graphical representation of the relationship between MMSE and Cognitive Prevalence (A), Motor Prevalence (B) and Physical Constraints (C) Components for the total sample (AD and age-matched HC) and for the two groups separately. Significance levels: ***: < .001; **: < .01; *: < .05.


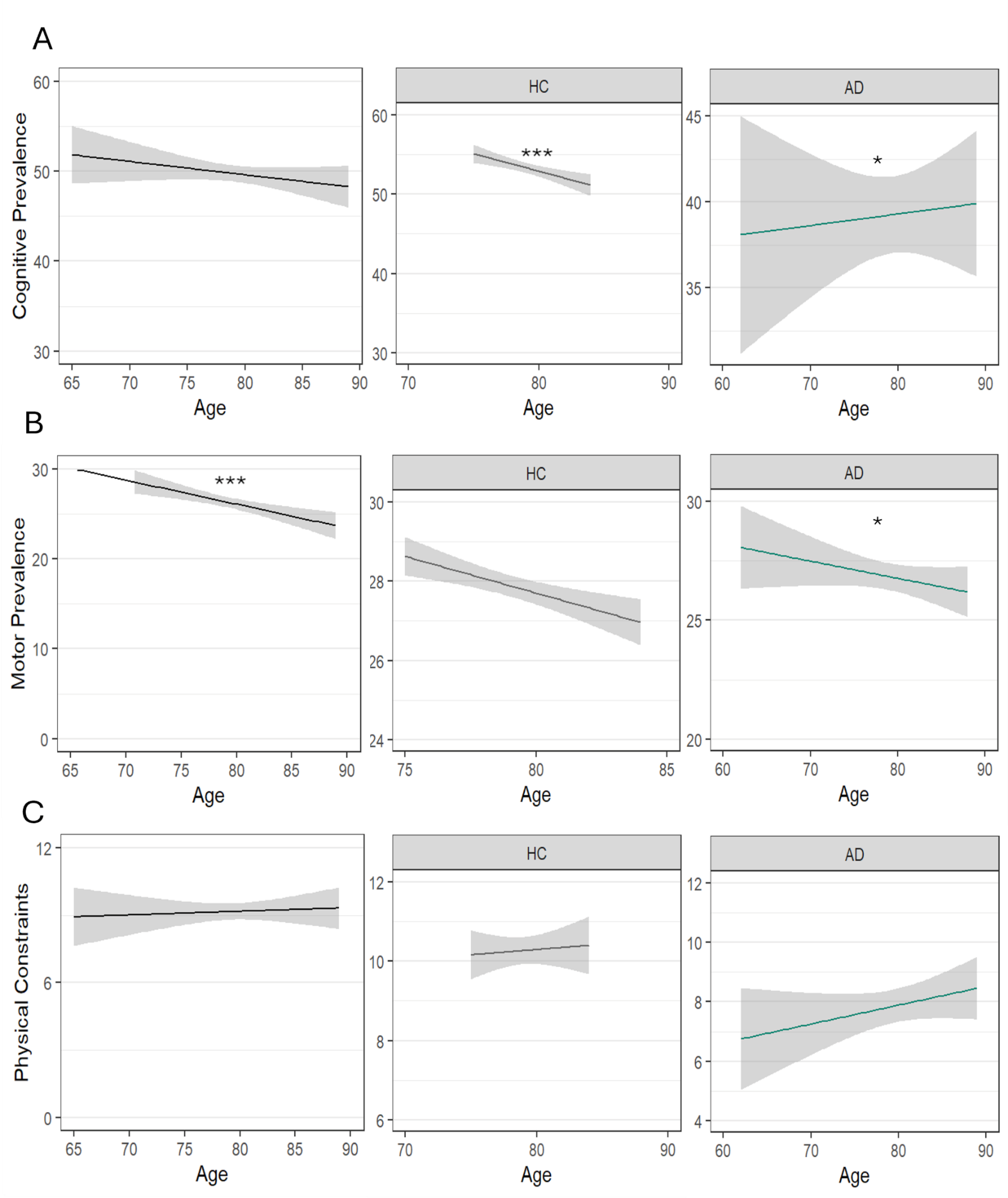


Figure SM5.2. Graphical representation of the relationship between Age and Cognitive Prevalence (A), Motor Prevalence (B) and Physical Constraints (C) Components for the total sample (AD and age-matched HC) and for the two groups separately. Significance levels: ***: < .001; **: < .01; *: < .05.

# SM6 – Internal Consistency, Test-Retest, Inter-Rater Reliability

The internal consistency and reliability of the EGP domains and components were investigated using the Cronbach alpha procedure on the total HC sample (n = 333), using the *alpha* function of R statistical software. Inter-rater consistency was calculated using Pearson’s correlation between the ratings provided by two independent examiners on a subsample of 29 healthy subjects (17 males, 12 Females; age: range: 66-93; M±SD:79.17±5.59). Internal consistency and Inter-Rater reliability are reported in the table below (Table SM6).

| **EGP Items** | **Cronbach α** | **Inter-Rater Reliability**  **(N= 29)** |
| --- | --- | --- |
| Static coordination I (M) | 0.88 | 1.00 |
| Static coordination II (M) | 0.88 | 1.00 |
| Dynamic coordination I (M) | 0.88 | 1.00 |
| Dynamic coordination II (M) | 0.88 | 1.00 |
| Joint mobilisation of upper limbs (Ph) | 0.89 | 0.98 |
| Joint mobilisation of lower limbs (Ph) | 0.89 | 1.00 |
| Hand fine motor skills (C) | 0.88 | 0.93 |
| Lower extremity fine motor skills (M) | 0.88 | 0.90 |
| Praxis (C) | 0.88 | 0.90 |
| Body representation (C) | 0.88 | 0.99 |
| Vigilance (C) | 0.89 | 0.94 |
| Perceptual Memory (C) | 0.88 | 0.99 |
| Space (C) | 0.88 | 0.96 |
| Verbal Memory (C) | 0.88 | 0.93 |
| Perception (C) | 0.88 | 0.87 |
| Temporal orientation (C) | 0.88 | 0.83 |
| Communication (C) | 0.89 | 0.99 |
| EGP – Total | 0.87 | 1.00 |
| Motor Prevalence | 0.88 | 1.00 |
| Cognitive Prevalence | 0.87 | 1.00 |
| Physical Constraints | 0.89 | 0.69 |

Table SM6: EGP Internal consistency and Inter-Rater reliability.

Moreover, Test-retest reliability was also calculated on a small subsample of healthy subjects using Pearson’s correlation coefficients. The test-retest sample consisted of 16 healthy participants (M=1; F=15) whose ages ranged from 66 to 85 years (M±SD: 75.94±6.02). The two EGP administrations were carried out with approximately a 1-month delay between them, and both the examiner and respondent were the same. Test-retest reliability was calculated for the EGP total score (0.60) and for the three components, i.e. Motor Prevalence (0.76), Cognitive Prevalence (0.59) and Physical Constraints (0.60).

# SM7 – Mean (SD), % Ceiling and % Floor on AD and HC total samples

In the table below (Table SM7), the means, standard deviations and the percentage of the ceiling and floor scores of the EGP items and components for both the total HC group and the AD sample.

|  | **HC (n=333)** | | | **AD (n= 89)** | | |
| --- | --- | --- | --- | --- | --- | --- |
| **Item** | **Mean (SD)** | **Ceiling (%)** | **Floor (%)** | **Mean (SD)** | **Ceiling (%)** | **Floor (%)** |
| Static coordination I (M) | 5.92 (0.45) | 95.8 | 0 | 5.73 (1.07) | 92.1 | 1.1 |
| Static coordination II (M) | 4.36 (1.38) | 20.1 | 2.7 | 3.28 (1.59) | 3.4 | 6.7 |
| Dynamic coordination I (M) | 5.86 (0.67) | 94.6 | 0 | 5.62 (1.20) | 88.8 | 2.2 |
| Dynamic coordination II (M) | 5.48 (1.38) | 85.9 | 3 | 4.63 (2.15) | 67.4 | 11.2 |
| Joint mobilisation of upper limbs (Ph) | 4.94 (1.21) | 44.4 | 0 | 3.73 (1.64) | 15.7 | 2.2 |
| Joint mobilisation of lower limbs (Ph) | 5.08 (1.22) | 51.4 | 0 | 3.84 (1.46) | 14.6 | 1.1 |
| Hand fine motor skills (C) | 5.36 (0.77) | 45.3 | 0 | 4.11 (1.42) | 12.4 | 1.1 |
| Lower extremity fine motor skills (M) | 5.86 (0.62) | 91.3 | 0.3 | 5.46 (1.07) | 71.9 | 0 |
| Praxis (C) | 5.17 (0.75) | 24.6 | 0 | 3.32 (1.41) | 2.2 | 3.4 |
| Body representation (C) | 5.46 (0.52) | 33.3 | 0 | 4.65 (1.42) | 22.5 | 2.2 |
| Vigilance (C) | 5.91 (0.28) | 89.5 | 0 | 5.13 (1.25) | 53.9 | 0 |
| Perceptual Memory (C) | 4.64 (1.11) | 20.4 | 0 | 2.20 (1.25) | 0 | 6.7 |
| Space (C) | 5.69 (0.54) | 64.6 | 0 | 4.02 (1.51) | 12.4 | 0 |
| Verbal Memory (C) | 5.14 (0.88) | 35.1 | 0 | 3.48 (1.50) | 10.1 | 3.4 |
| Perception (C) | 5.31 (0.74) | 34.2 | 0 | 4.09 (1.28) | 9 | 0 |
| Temporal orientation (C) | 5.68 (0.5) | 61.3 | 0 | 3.40 (1.51) | 3.4 | 4.5 |
| Communication (C) | 5.91 (0.43) | 94.3 | 0 | 4.87 (1.59) | 58.4 | 0 |
| EGP – Total | 91.77 (6.93) | 0 | 0 | 71.55 (14.03) | 0 | 0 |
| Motor Prevalence | 27.48 (3.63) | 18.6 | 0 | 24.72 (5.72) | 3.4 | 0 |
| Cognitive Prevalence | 54.27 (3.88) | 1.5 | 0 | 39.26 (7.42) | 0 | 0 |
| Physical Constraints | 10.02 (2.24) | 33 | 0 | 7.57 (2.88) | 10.1 | 1.1 |

Table SM7: Percentage of maximum (%Ceiling) and minimum (%Floor) scores for EGP single items and for the three components in the healthy control sample (n = 333).
